# Supplementary material for: Maximizing insights from longitudinal epigenetic age data: simulations, applications, and practical guidance
Source: Clin Epigenetics. 2024 Dec 20;16:187. doi: 10.1186/s13148-024-01784-x (PMC11662605; doi:10.1186/s13148-024-01784-x)
Supplement: Supplementary file 1 — Additional file 1. [file 13148_2024_1784_MOESM1_ESM.pdf]

Supplemental Materials

Maximising Insights from Longitudinal Epigenetic Age Data: Simulations, Applications, and Practical Guidance

Two Measure-based Simulation

Table 1: Summary of fixed effect estimates and 95% confidence intervals (Binary Exposure)

Table 2: Summary of interaction effect estimates and 95% confidence intervals (Binary Exposure)

Table 3: Summary of fixed effect estimates and 95% confidence intervals (Continuous Exposure)

Table 4: Summary of interaction effect estimates and 95% confidence intervals (Continuous Exposure)

Figure 1: Binary exposure simulation results (Horvath)

Figure 2: Continuous exposure simulation results (Horvath)

Figure 3: Binary exposure simulation results (GrimAge)

Figure 4: Continuous exposure simulation results (GrimAge)

Figure 5: Binary exposure simulation results (PC Horvath)

Figure 6: Continuous exposure simulation results (PC Horvath)

Figure 7: Binary exposure simulation results (PC GrimAge)

Figure 8: Continuous exposure simulation results (PC GrimAge)

Figure 9: Evaluation of confidence intervals from binary exposure simulation (Horvath)

Figure 10: Evaluation of confidence intervals from continuous exposure simulation (Horvath)

Figure 11: Evaluation of confidence intervals from binary exposure simulation (GrimAge)

Figure 12: Evaluation of confidence intervals from continuous exposure simulation (GrimAge)

Figure 13: Evaluation of confidence intervals from binary exposure simulation (PC Horvath)

Figure 14: Evaluation of confidence intervals from continuous exposure simulation (PC Horvath)

Figure 15: Evaluation of confidence intervals from binary exposure simulation (PC GrimAge)

Figure 16: Evaluation of confidence intervals from continuous exposure simulation (PC GrimAge)

Three Measure-based Simulation

Table 5: Summary of fixed effect estimates and 95% confidence intervals

Table 6: Summary of interaction effect estimates and 95% confidence intervals

Figure 17: Binary exposure simulation results (Horvath)

Figure 18: Continuous exposure simulation results (Horvath)

Figure 19: Binary exposure simulation results (GrimAge)

Figure 20: Continuous exposure simulation results (GrimAge)

Figure 21: Evaluation of confidence intervals from binary exposure simulation (Horvath)

Figure 22: Evaluation of confidence intervals from continuous exposure simulation (Horvath)

Figure 23: Evaluation of confidence intervals from binary exposure simulation (GrimAge)

Figure 24: Evaluation of confidence intervals from continuous exposure simulation (GrimAge)

Real-world Example

Table 7: Fixed effect estimates from biological sex on longitudinal EA

Table 8: Interaction effect estimates from biological sex on longitudinal EA

Table 9: Fixed effect estimates from birthweight on longitudinal EA

Table 10: Interaction effect estimates from birthweight on longitudinal EA

Figure 25: Fixed effect estimates of biological sex on offspring EA over time

Maximising Insights from Longitudinal Epigenetic Age Data: Simulations, Applications, and Practical Guidance

Figure 26: Age interaction estimates of biological age on offspring EA over time
Figure 27: Fixed effect estimates of birthweight on offspring EA over time
Figure 28: Age interaction estimates of birthweight on offspring EA over time

1. Two Measure-based Simulation

***Table 1**: Summary of fixed effect estimates and confidence intervals (CI) from simulations (n = 1,000) including a binary exposure variable (effect size: 2), based on ARIES cohort data (two measurements, age 7 and 15-17)* [1]. *Methods used to model the simulated exposures differed in (i) the type of model, (ii) the outcome and (iii) time variables included in the model. Rows show results from each model and time variable. Columns divide results in outcome variables included in the model. Models are Linear Mixed Effect Models (LME), Generalized Estimating Equations (GEE), and regression on difference between two epigenetic age (EA) measures (Δ aging). Time variables are chronological age (Age), years between measures (Years), number of measure (Timepoint, i.e., 1, 2, 3), factorized measure (Timefactor, i.e., F07, F09, F15). Outcome variables are EAA (i.e., residual from regressing EA on age), or EA itself, derived from either the Horvath clock* [2], *GrimAge* [3], *or their principal component versions* [4]. *We calculated the CI as mean estimate ± mean standard error across all simulations.*

Model	Time Variable	Outcome							
		Horvath		GrimAge		PC Horvath		PC GrimAge	
		EA	EAA	EA	EAA	EA	EAA	EA	EAA
		**Fixed Effect** Estimate (CI)		**Fixed Effect** Estimate (CI)		**Fixed Effect** Estimate (CI)		**Fixed Effect** Estimate (CI)	
LME	Age	2.02 (0.73,3.31)	2.02 (0.73,3.31)	2.01 (1.14,2.89)	2.01 (1.15,2.88)	1.98 (0.85,3.12)	1.98 (0.85,3.12)	2.01 (1.24,2.77)	2 (1.24,2.76)
	Years	2.65 (1.86,3.44)	2.65 (1.86,3.44)	2.65 (2.09,3.21)	2.65 (2.1,3.2)	2.62 (1.87,3.38)	2.62 (1.87,3.37)	2.64 (2.13,3.15)	2.64 (2.14,3.14)
	Timepoint	1.81 (0.28,3.34)	1.81 (0.31,3.3)	1.79 (0.72,2.87)	1.79 (0.8,2.79)	1.77 (0.43,3.1)	1.76 (0.47,3.06)	1.79 (0.82,2.76)	1.78 (0.91,2.65)
	Timefactor	3.22 (2.65,3.79)	3.22 (2.66,3.78)	3.23 (2.8,3.66)	3.23 (2.81,3.64)	3.21 (2.59,3.82)	3.2 (2.61,3.8)	3.22 (2.8,3.64)	3.21 (2.82,3.61)
LME (random slope)	Age	2.02 (1,3.04)	2.02 (1,3.04)	2.01 (1.18,2.84)	2.01 (1.18,2.84)	1.98 (1.09,2.88)	1.98 (1.09,2.88)	2.01 (1.25,2.76)	2 (1.25,2.75)
	Years	2.65 (2.09,3.22)	2.65 (2.08,3.21)	2.65 (2.13,3.17)	2.65 (2.13,3.17)	2.62 (2.04,3.2)	2.62 (2.05,3.2)	2.64 (2.14,3.14)	2.64 (2.14,3.13)
	Timepoint	1.81 (0.58,3.04)	1.81 (0.59,3.02)	1.79 (0.8,2.79)	1.79 (0.84,2.75)	1.77 (0.72,2.81)	1.76 (0.72,2.8)	1.79 (0.89,2.69)	1.78 (0.92,2.64)
	Timefactor	3.22 (2.65,3.79)	3.22 (2.66,3.78)	3.23 (2.8,3.66)	3.23 (2.81,3.64)	3.21 (2.59,3.82)	3.2 (2.61,3.8)	3.22 (2.8,3.64)	3.21 (2.82,3.61)
GEE	Age	2.02 (0.87,3.17)	2.02 (0.87,3.17)	2.02 (1.07,2.97)	2.01 (1.06,2.96)	1.98 (0.83,3.14)	1.98 (0.83,3.13)	2.01 (1.11,2.9)	2 (1.11,2.89)
	Years	2.65 (2.07,3.24)	2.65 (2.06,3.23)	2.65 (2.11,3.19)	2.65 (2.11,3.19)	2.62 (2.01,3.24)	2.62 (2.01,3.23)	2.64 (2.13,3.15)	2.64 (2.12,3.15)
	Timepoint	1.81 (0.42,3.19)	1.81 (0.44,3.17)	1.79 (0.66,2.93)	1.79 (0.68,2.9)	1.76 (0.36,3.17)	1.76 (0.39,3.13)	1.79 (0.7,2.87)	1.78 (0.74,2.82)
	Timefactor	3.22 (2.7,3.75)	3.22 (2.71,3.73)	3.23 (2.85,3.61)	3.23 (2.87,3.59)	3.21 (2.69,3.72)	3.2 (2.71,3.7)	3.22 (2.85,3.58)	3.21 (2.88,3.54)
**Exposure**		Binary Exposure, Simulated Fixed Effect: **2.0**							

1. Two Measure-based Simulation

***Table 2**: Summary of interaction effect estimates and confidence intervals (CI) from simulations (n = 1,000) including a binary exposure variable (effect size: 0.1), based on ARIES cohort data (two measurements, age 7 and 15-17)* [1]. *Methods used to model the simulated exposures differed in (i) the type of model, (ii) the outcome and (iii) time variables included in the model. Rows show results from each model and time variable. Columns divide results in outcome variables included in the model. Models are Linear Mixed Effect Models (LME), Generalized Estimating Equations (GEE), and regression on difference between two epigenetic age (EA) measures (Δ aging). Time variables are chronological age (Age), years between measures (Years), number of measure (Timepoint, i.e., 1, 2, 3), factorized measure (Timefactor, i.e., F07, F09, F15). Outcome variables are EAA (i.e., residual from regressing EA on age), or EA itself, derived from either the Horvath clock* [2], *GrimAge* [3], *or their principal component versions* [4]. *We calculated the CI as mean estimate ± mean standard error across all simulations.*

**Model**	**Time Variable**	**Outcome**							
		Horvath		GrimAge		PC Horvath		PC GrimAge	
		EA	EAA	EA	EAA	EA	EAA	EA	EAA
		**Interaction** Estimate (CI)		**Interaction** Estimate (CI)		**Interaction** Estimate (CI)		**Interaction** Estimate (CI)	
LME	Age	0.1 (0,0.19)	0.1 (0,0.19)	0.1 (0.04,0.16)	0.1 (0.04,0.16)	0.1 (0.02,0.18)	0.1 (0.02,0.18)	0.1 (0.05,0.15)	0.1 (0.05,0.15)
	Years	0.1 (0,0.2)	0.1 (0,0.2)	0.1 (0.04,0.17)	0.1 (0.04,0.16)	0.1 (0.02,0.18)	0.1 (0.02,0.18)	0.1 (0.04,0.16)	0.1 (0.05,0.15)
	Timepoint	0.94 (0,1.89)	0.94 (0.02,1.87)	0.96 (0.3,1.61)	0.96 (0.35,1.56)	0.96 (0.17,1.75)	0.96 (0.19,1.73)	0.95 (0.37,1.54)	0.95 (0.44,1.47)
	Timefactor	0.67 (0,1.34)	0.67 (0.01,1.32)	0.68 (0.21,1.14)	0.68 (0.25,1.1)	0.68 (0.12,1.24)	0.68 (0.14,1.22)	0.67 (0.26,1.09)	0.67 (0.31,1.04)
LME (random slope)	Age	0.1 (0.01,0.18)	0.1 (0.01,0.18)	0.1 (0.04,0.16)	0.1 (0.04,0.16)	0.1 (0.02,0.18)	0.1 (0.02,0.18)	0.1 (0.05,0.15)	0.1 (0.05,0.15)
	Years	0.1 (0,0.2)	0.1 (0,0.2)	0.1 (0.04,0.17)	0.1 (0.04,0.16)	0.1 (0.02,0.18)	0.1 (0.02,0.18)	0.1 (0.04,0.16)	0.1 (0.05,0.15)
	Timepoint	0.94 (0.1,1.78)	0.94 (0.11,1.77)	0.96 (0.3,1.61)	0.96 (0.35,1.56)	0.96 (0.21,1.71)	0.96 (0.22,1.7)	0.95 (0.37,1.54)	0.95 (0.44,1.47)
	Timefactor	0.67 (0,1.34)	0.67 (0.01,1.32)	0.68 (0.21,1.14)	0.68 (0.25,1.1)	0.68 (0.12,1.24)	0.68 (0.14,1.22)	0.67 (0.26,1.09)	0.67 (0.31,1.04)
GEE	Age	0.1 (-0.01,0.2)	0.1 (-0.01,0.2)	0.1 (0.02,0.17)	0.1 (0.02,0.17)	0.1 (0,0.2)	0.1 (0,0.2)	0.1 (0.03,0.17)	0.1 (0.03,0.17)
	Years	0.1 (-0.01,0.21)	0.1 (-0.01,0.21)	0.1 (0.02,0.18)	0.1 (0.02,0.18)	0.1 (0,0.21)	0.1 (0,0.21)	0.1 (0.03,0.17)	0.1 (0.03,0.17)
	Timepoint	0.94 (-0.1,1.99)	0.94 (-0.08,1.97)	0.96 (0.19,1.72)	0.96 (0.24,1.68)	0.96 (-0.07,1.99)	0.96 (-0.03,1.95)	0.95 (0.23,1.68)	0.95 (0.29,1.62)
	Timefactor	0.67 (-0.07,1.41)	0.67 (-0.06,1.39)	0.68 (0.14,1.22)	0.68 (0.17,1.19)	0.68 (-0.05,1.41)	0.68 (-0.02,1.38)	0.67 (0.16,1.19)	0.67 (0.21,1.14)
Δ aging	Age	0.1 (0,0.2)		0.1 (0.04,0.16)		0.1 (0.02,0.18)		0.1 (0.04,0.15)	
	None	0.94 (-0.02,1.9)	0.94 (0,1.88)	0.96 (0.3,1.62)	0.96 (0.35,1.56)	0.96 (0.16,1.76)	0.96 (0.19,1.73)	0.95 (0.37,1.54)	0.95 (0.44,1.47)
**Exposure**		Binary Exposure, Simulated Interaction Effect: **0.1**							

1. Two Measure-based Simulation

***Table 3**: Summary of fixed effect estimates and confidence intervals (CI) from simulations (n = 1,000) including a continuous exposure variable (effect size: 0.1), based on ARIES cohort data (two measurements, age 7 and 15-17)* [1]. *Methods used to model the simulated exposures differed in (i) the type of model, (ii) the outcome and (iii) time variables included in the model. Rows show results from each model and time variable. Columns divide results in outcome variables included in the model. Models are Linear Mixed Effect Models (LME), Generalized Estimating Equations (GEE), and regression on difference between two epigenetic age (EA) measures (Δ aging). Time variables are chronological age (Age), years between measures (Years), number of measure (Timepoint, i.e., 1, 2, 3), factorized measure (Timefactor, i.e., F07, F09, F15). Outcome variables are EAA (i.e., residual from regressing EA on age), or EA itself, derived from either the Horvath clock* [2], *GrimAge* [3], *or their principal component versions* [4]. *We calculated the CI as mean estimate ± mean standard error across all simulations.*

**Model**	**Time Variable**	**Outcome**							
		Horvath		GrimAge		PC Horvath		PC GrimAge	
		EA	EAA	EA	EAA	EA	EAA	EA	EAA
		**Fixed Effect** Estimate (CI)		**Fixed Effect** Estimate (CI)		**Fixed Effect** Estimate (CI)		**Fixed Effect** Estimate (CI)	
LME	Age	0.11 (-0.67,0.88)	0.11 (-0.67,0.88)	0.09 (-0.43,0.62)	0.09 (-0.43,0.61)	0.1 (-0.58,0.78)	0.1 (-0.58,0.78)	0.1 (-0.36,0.56)	0.1 (-0.36,0.56)
	Years	0.24 (-0.24,0.71)	0.24 (-0.24,0.71)	0.23 (-0.11,0.56)	0.23 (-0.1,0.56)	0.23 (-0.22,0.68)	0.23 (-0.22,0.68)	0.23 (-0.08,0.54)	0.23 (-0.07,0.53)
	Timepoint	0.07 (-0.86,0.99)	0.07 (-0.83,0.96)	0.05 (-0.6,0.7)	0.05 (-0.55,0.65)	0.05 (-0.75,0.86)	0.06 (-0.72,0.83)	0.06 (-0.53,0.65)	0.06 (-0.46,0.58)
	Timefactor	0.35 (0.01,0.7)	0.35 (0.02,0.69)	0.35 (0.09,0.61)	0.35 (0.1,0.6)	0.35 (-0.02,0.72)	0.35 (-0.01,0.7)	0.35 (0.09,0.6)	0.34 (0.11,0.58)
LME (random slope)	Age	0.11 (-0.5,0.72)	0.11 (-0.5,0.72)	0.09 (-0.4,0.59)	0.09 (-0.4,0.59)	0.1 (-0.44,0.64)	0.1 (-0.44,0.64)	0.1 (-0.35,0.55)	0.1 (-0.35,0.55)
	Years	0.24 (-0.1,0.58)	0.24 (-0.1,0.58)	0.23 (-0.08,0.54)	0.23 (-0.08,0.54)	0.23 (-0.12,0.58)	0.23 (-0.12,0.58)	0.23 (-0.07,0.53)	0.23 (-0.07,0.53)
	Timepoint	0.06 (-0.67,0.8)	0.07 (-0.66,0.79)	0.05 (-0.55,0.65)	0.05 (-0.53,0.62)	0.05 (-0.58,0.68)	0.06 (-0.57,0.68)	0.06 (-0.48,0.6)	0.06 (-0.46,0.58)
	Timefactor	0.35 (0.01,0.7)	0.35 (0.02,0.69)	0.35 (0.09,0.61)	0.35 (0.1,0.6)	0.35 (-0.02,0.72)	0.35 (-0.01,0.7)	0.35 (0.09,0.6)	0.34 (0.11,0.58)
GEE	Age	0.11 (-0.58,0.8)	0.11 (-0.58,0.8)	0.09 (-0.48,0.66)	0.09 (-0.48,0.66)	0.1 (-0.6,0.79)	0.1 (-0.6,0.79)	0.1 (-0.44,0.64)	0.1 (-0.44,0.64)
	Years	0.24 (-0.12,0.59)	0.24 (-0.12,0.59)	0.23 (-0.1,0.55)	0.23 (-0.1,0.55)	0.23 (-0.14,0.6)	0.23 (-0.14,0.6)	0.23 (-0.08,0.54)	0.23 (-0.08,0.54)
	Timepoint	0.07 (-0.77,0.9)	0.07 (-0.76,0.89)	0.05 (-0.63,0.73)	0.05 (-0.62,0.71)	0.05 (-0.8,0.9)	0.05 (-0.78,0.88)	0.06 (-0.59,0.71)	0.06 (-0.57,0.69)
	Timefactor	0.35 (0.04,0.67)	0.35 (0.04,0.66)	0.35 (0.12,0.58)	0.35 (0.13,0.57)	0.35 (0.04,0.66)	0.35 (0.05,0.65)	0.35 (0.13,0.56)	0.34 (0.15,0.54)
**Exposure**		Continuous Exposure, Simulated Fixed Effect: **0.1**							

1. Two Measure-based Simulation

***Table 4**: Summary of interaction effect estimates and confidence intervals (CI) from simulations (n = 1,000) including a continuous exposure variable (effect size: 0.02), based on ARIES cohort data (two measurements, age 7 and 15-17)* [1]*. Methods used to model the simulated exposures differed in (i) the type of model, (ii) the outcome and (iii) time variables included in the model. Rows show results from each model and time variable. Columns divide results in outcome variables included in the model. Models are Linear Mixed Effect Models (LME), Generalized Estimating Equations (GEE), and regression on difference between two epigenetic age (EA) measures (Δ aging). Time variables are chronological age (Age), years between measures (Years), number of measure (Timepoint, i.e., 1, 2, 3), factorized measure (Timefactor, i.e., F07, F09, F15). Outcome variables are EAA (i.e., residual from regressing EA on age), or EA itself, derived from either the Horvath clock* [2], *GrimAge* [3], *or their principal component versions* [4]. *We calculated the CI as mean estimate ± mean standard error across all simulations.*

**Model**	**Time Variable**	**Outcome**							
		Horvath		GrimAge		PC Horvath		PC GrimAge	
		EA	EAA	EA	EAA	EA	EAA	EA	EAA
		**Interaction** Estimate (CI)		**Interaction** Estimate (CI)		**Interaction** Estimate (CI)		**Interaction** Estimate (CI)	
LME	Age	0.02 (-0.04,0.08)	0.02 (-0.04,0.08)	0.02 (-0.02,0.06)	0.02 (-0.02,0.06)	0.02 (-0.03,0.07)	0.02 (-0.03,0.07)	0.02 (-0.01,0.05)	0.02 (-0.01,0.05)
	Years	0.02 (-0.04,0.08)	0.02 (-0.04,0.08)	0.02 (-0.02,0.06)	0.02 (-0.02,0.06)	0.02 (-0.03,0.07)	0.02 (-0.03,0.07)	0.02 (-0.01,0.05)	0.02 (-0.01,0.05)
	Timepoint	0.19 (-0.38,0.76)	0.19 (-0.36,0.75)	0.2 (-0.2,0.6)	0.2 (-0.16,0.56)	0.2 (-0.28,0.67)	0.19 (-0.27,0.65)	0.19 (-0.16,0.55)	0.19 (-0.12,0.5)
	Timefactor	0.14 (-0.27,0.54)	0.13 (-0.26,0.53)	0.14 (-0.14,0.42)	0.14 (-0.11,0.4)	0.14 (-0.2,0.48)	0.14 (-0.19,0.46)	0.14 (-0.12,0.39)	0.14 (-0.08,0.35)
LME (random slope)	Age	0.02 (-0.03,0.07)	0.02 (-0.03,0.07)	0.02 (-0.02,0.06)	0.02 (-0.02,0.06)	0.02 (-0.03,0.07)	0.02 (-0.03,0.07)	0.02 (-0.01,0.05)	0.02 (-0.01,0.05)
	Years	0.02 (-0.04,0.08)	0.02 (-0.04,0.08)	0.02 (-0.02,0.06)	0.02 (-0.02,0.06)	0.02 (-0.03,0.07)	0.02 (-0.03,0.07)	0.02 (-0.01,0.05)	0.02 (-0.01,0.05)
	Timepoint	0.19 (-0.31,0.7)	0.19 (-0.31,0.69)	0.2 (-0.2,0.6)	0.2 (-0.16,0.56)	0.2 (-0.26,0.65)	0.19 (-0.25,0.64)	0.19 (-0.16,0.55)	0.19 (-0.12,0.5)
	Timefactor	0.14 (-0.27,0.54)	0.13 (-0.26,0.53)	0.14 (-0.14,0.42)	0.14 (-0.11,0.4)	0.14 (-0.2,0.48)	0.14 (-0.19,0.46)	0.14 (-0.12,0.39)	0.14 (-0.08,0.35)
GEE	Age	0.02 (-0.04,0.08)	0.02 (-0.04,0.08)	0.02 (-0.02,0.07)	0.02 (-0.02,0.07)	0.02 (-0.04,0.08)	0.02 (-0.04,0.08)	0.02 (-0.02,0.06)	0.02 (-0.02,0.06)
	Years	0.02 (-0.05,0.09)	0.02 (-0.05,0.09)	0.02 (-0.03,0.07)	0.02 (-0.02,0.07)	0.02 (-0.04,0.08)	0.02 (-0.04,0.08)	0.02 (-0.02,0.06)	0.02 (-0.02,0.06)
	Timepoint	0.19 (-0.44,0.82)	0.19 (-0.42,0.81)	0.2 (-0.26,0.66)	0.2 (-0.23,0.63)	0.2 (-0.42,0.82)	0.2 (-0.4,0.8)	0.19 (-0.25,0.63)	0.19 (-0.21,0.59)
	Timefactor	0.14 (-0.31,0.58)	0.13 (-0.3,0.57)	0.14 (-0.18,0.47)	0.14 (-0.16,0.45)	0.14 (-0.3,0.58)	0.14 (-0.29,0.56)	0.14 (-0.17,0.44)	0.13 (-0.15,0.42)
Δ aging	Age	0.02 (-0.04,0.08)		0.02 (-0.02,0.06)		0.02 (-0.03,0.07)		0.02 (-0.01,0.05)	
	None	0.19 (-0.38,0.77)	0.19 (-0.37,0.75)	0.2 (-0.2,0.6)	0.2 (-0.16,0.56)	0.19 (-0.29,0.67)	0.19 (-0.27,0.66)	0.19 (-0.16,0.55)	0.19 (-0.12,0.5)
**Exposure**		Continuous Exposure, Simulated Interaction Effect: **0.02**							

1. Two Measure-based Simulation

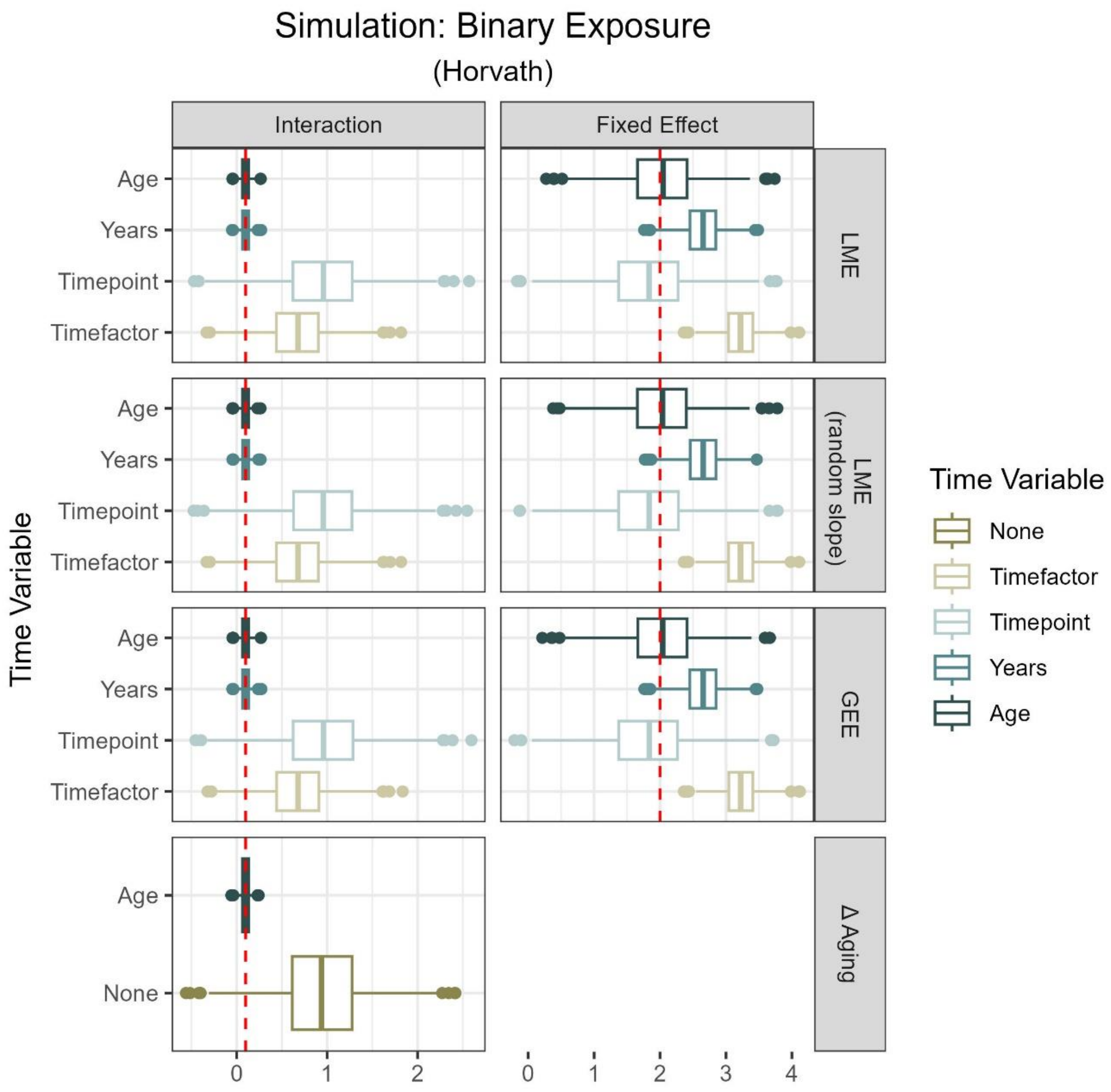

***Figure 1**: Binary exposure simulation results (n = 1,000) based on longitudinal ARIES data (two measurements, age 7 and 15-17)* [1]*. Rows show boxplots of effect size estimates derived from different models and time variables included in those models respectively. The two columns divide into interaction effect (left column) and fixed effect (right column). Simulated interaction effect of 0.1, and simulated fixed effect of 2, are marked in red. Models are Linear Mixed Effect Models (LME), Generalized Estimating Equations (GEE), and regression on difference between two epigenetic age (EA) measures (Δ aging). Time variables are chronological age (Age), years between measures (Years), number of measure (Timepoint, i.e., 1, 2, 3), factorized measure (Timefactor, i.e., F07, F09, F15). All models contained Horvath clock* [2] *derived EA as outcome.*

1. Two Measure-based Simulation

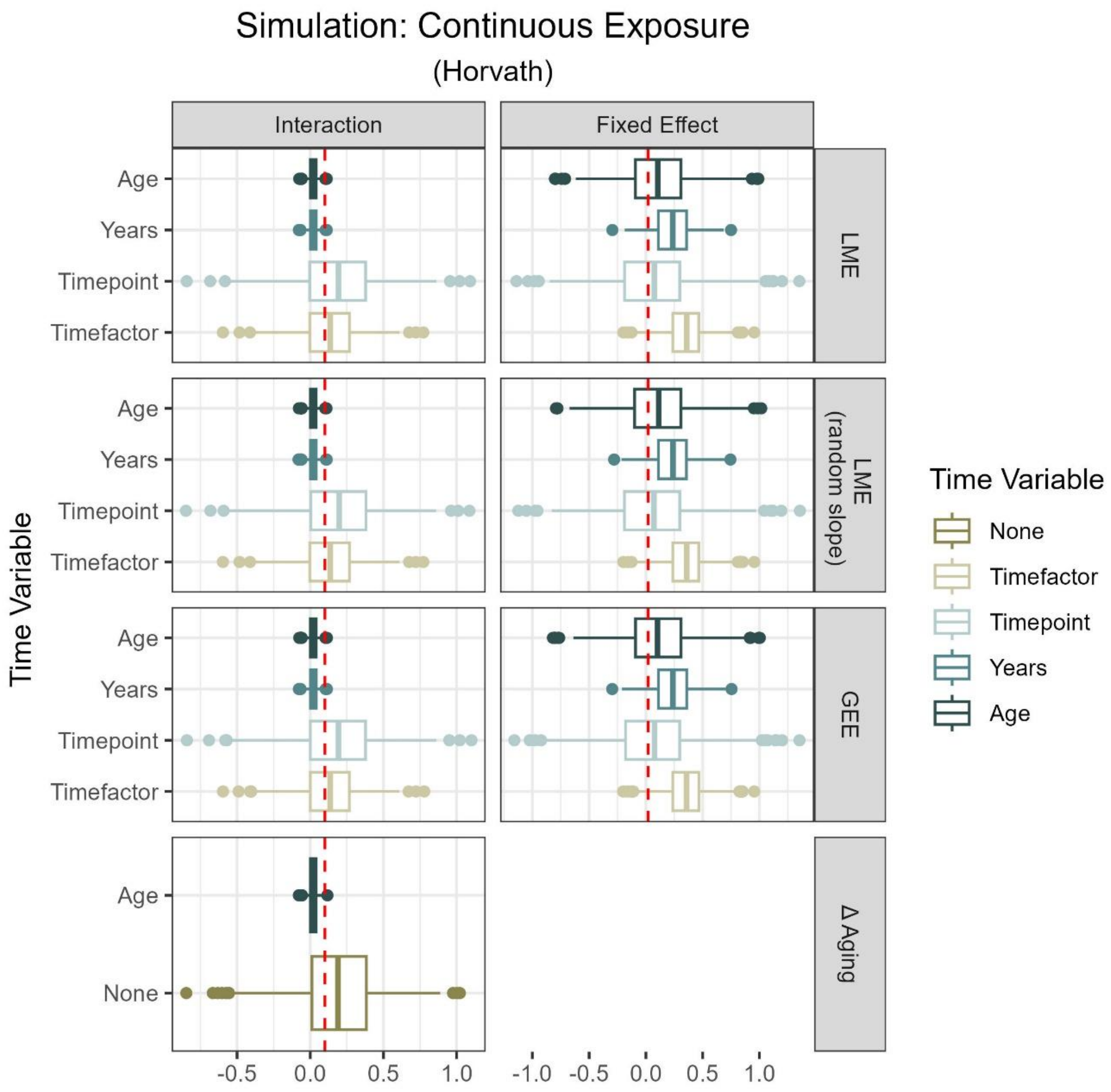

***Figure 2**: Continuous exposure simulation results (n = 1,000) based on longitudinal ARIES data (two measurements, age 7 and 15-17)* [1]*. Rows show boxplots of effect size estimates derived from different models and time variables included in those models respectively. The two columns divide into interaction effect (left column) and fixed effect (right column). Simulated interaction effect of 0.02, and simulated fixed effect of 0.1, are marked in red. Models are Linear Mixed Effect Models (LME), Generalized Estimating Equations (GEE), and regression on difference between two epigenetic age (EA) measures (Δ aging). Time variables are chronological age (Age), years between measures (Years), number of measure (Timepoint, i.e., 1, 2, 3), factorized measure (Timefactor, i.e., F07, F09, F15). All models contained Horvath clock* [2] *derived EA as outcome.*

1. Two Measure-based Simulation

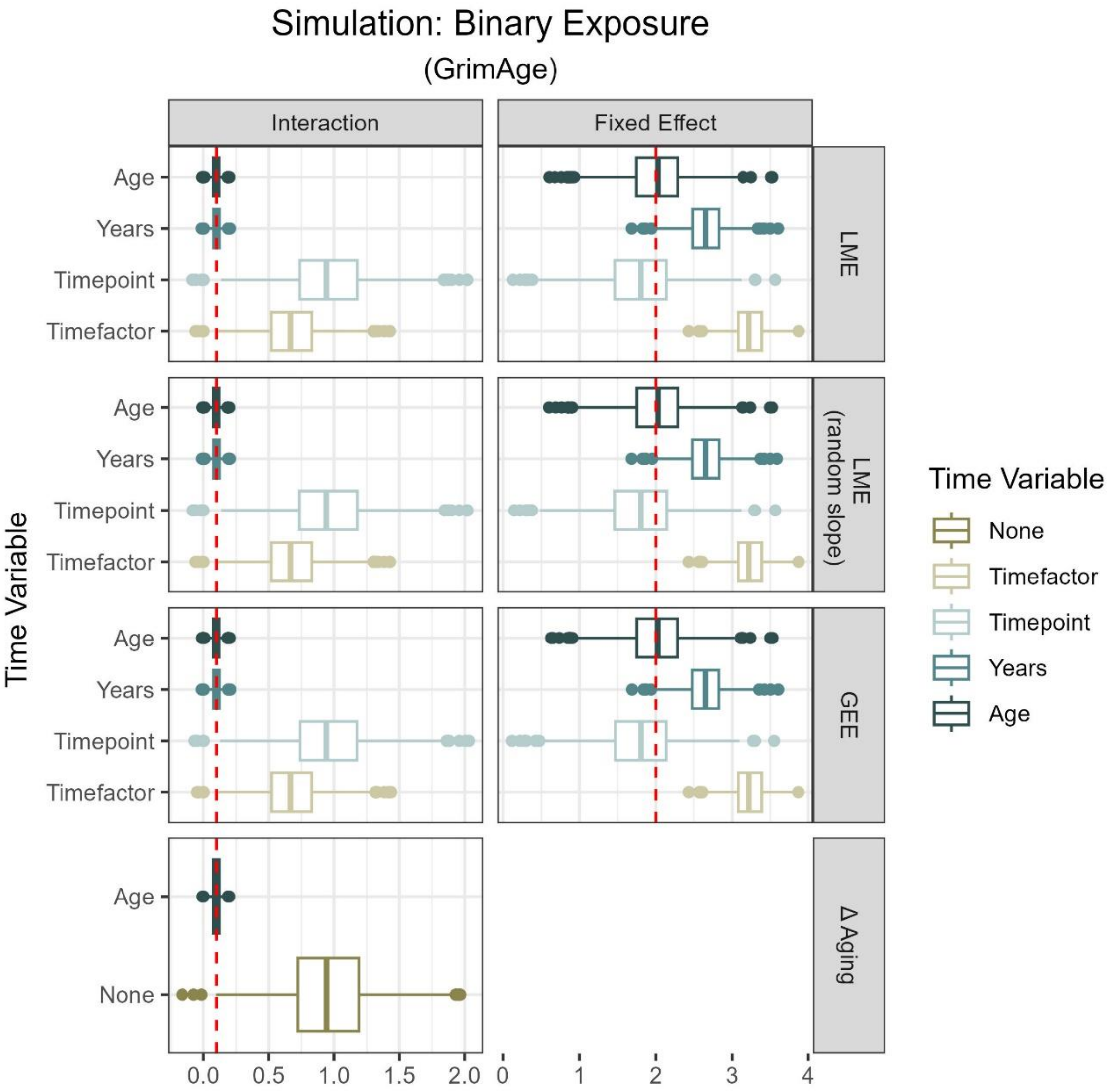

***Figure 3**: Binary exposure simulation results (n = 1,000) based on longitudinal ARIES data (two measurements, age 7 and 15-17)* [1]. *Rows show boxplots of effect size estimates derived from different models and time variables included in those models respectively. The two columns divide into interaction effect (left column) and fixed effect (right column). Simulated interaction effect of 0.1, and simulated fixed effect of 2, are marked in red. Models are Linear Mixed Effect Models (LME), Generalized Estimating Equations (GEE), and regression on difference between two epigenetic age (EA) measures (Δ aging). Time variables are chronological age (Age), years between measures (Years), number of measure (Timepoint, i.e., 1, 2, 3), factorized measure (Timefactor, i.e., F07, F09, F15). All models contained GrimAge* [3] *derived EA as outcome.*

1. Two Measure-based Simulation

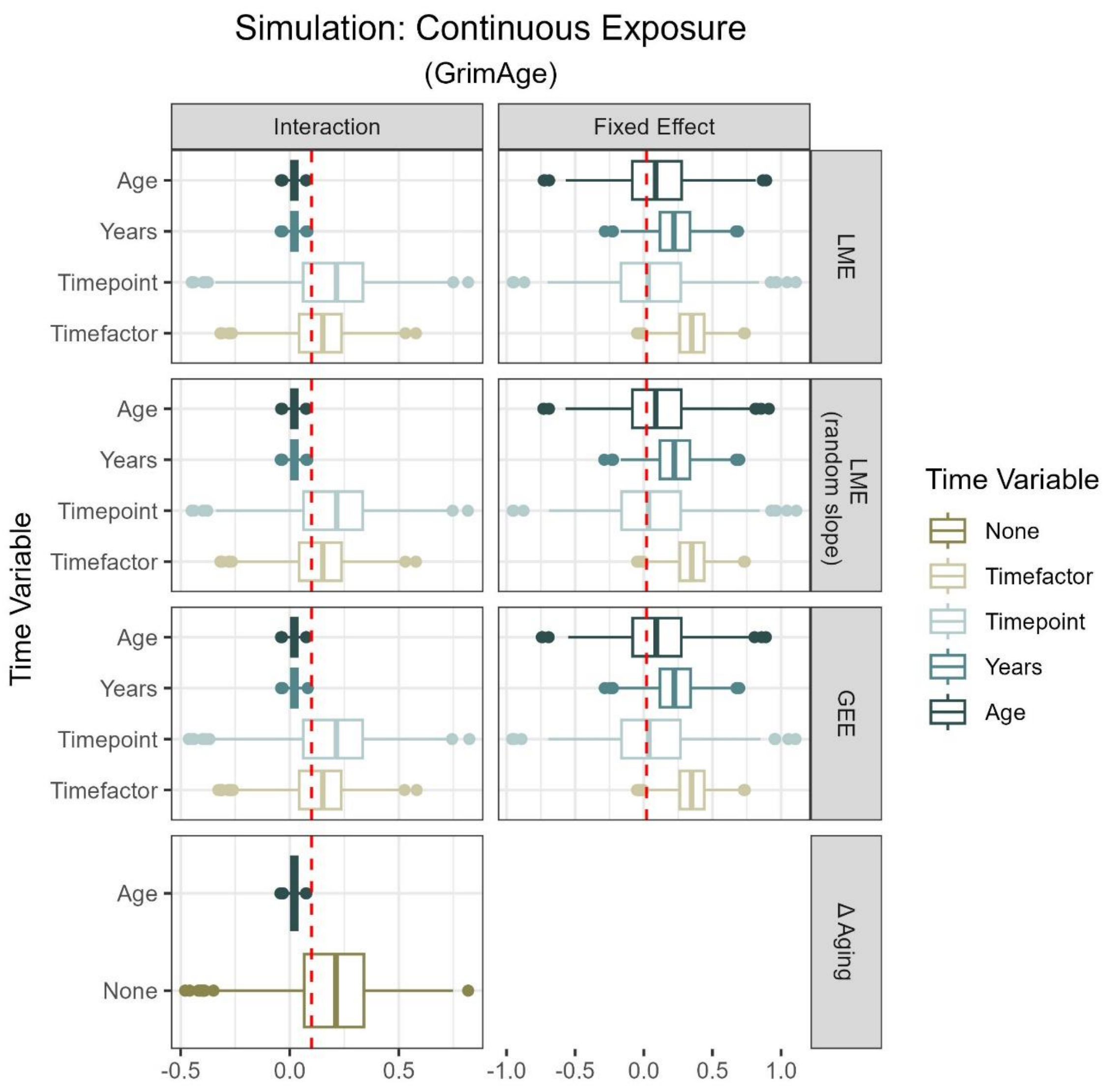

***Figure 4**: Continuous exposure simulation results (n = 1,000) based on longitudinal ARIES data (two measurements, age 7 and 15-17)* [1]*. Rows show boxplots of effect size estimates derived from different models and time variables included in those models respectively. The two columns divide into interaction effect (left column) and fixed effect (right column). Simulated interaction effect of 0.02, and simulated fixed effect of 0.1, are marked in red. Models are Linear Mixed Effect Models (LME), Generalized Estimating Equations (GEE), and regression on difference between two epigenetic age (EA) measures (Δ aging). Time variables are chronological age (Age), years between measures (Years), number of measure (Timepoint, i.e., 1, 2, 3), factorized measure (Timefactor, i.e., F07, F09, F15). All models contained GrimAge* [3] *derived EA as outcome.*

1. Two Measure-based Simulation

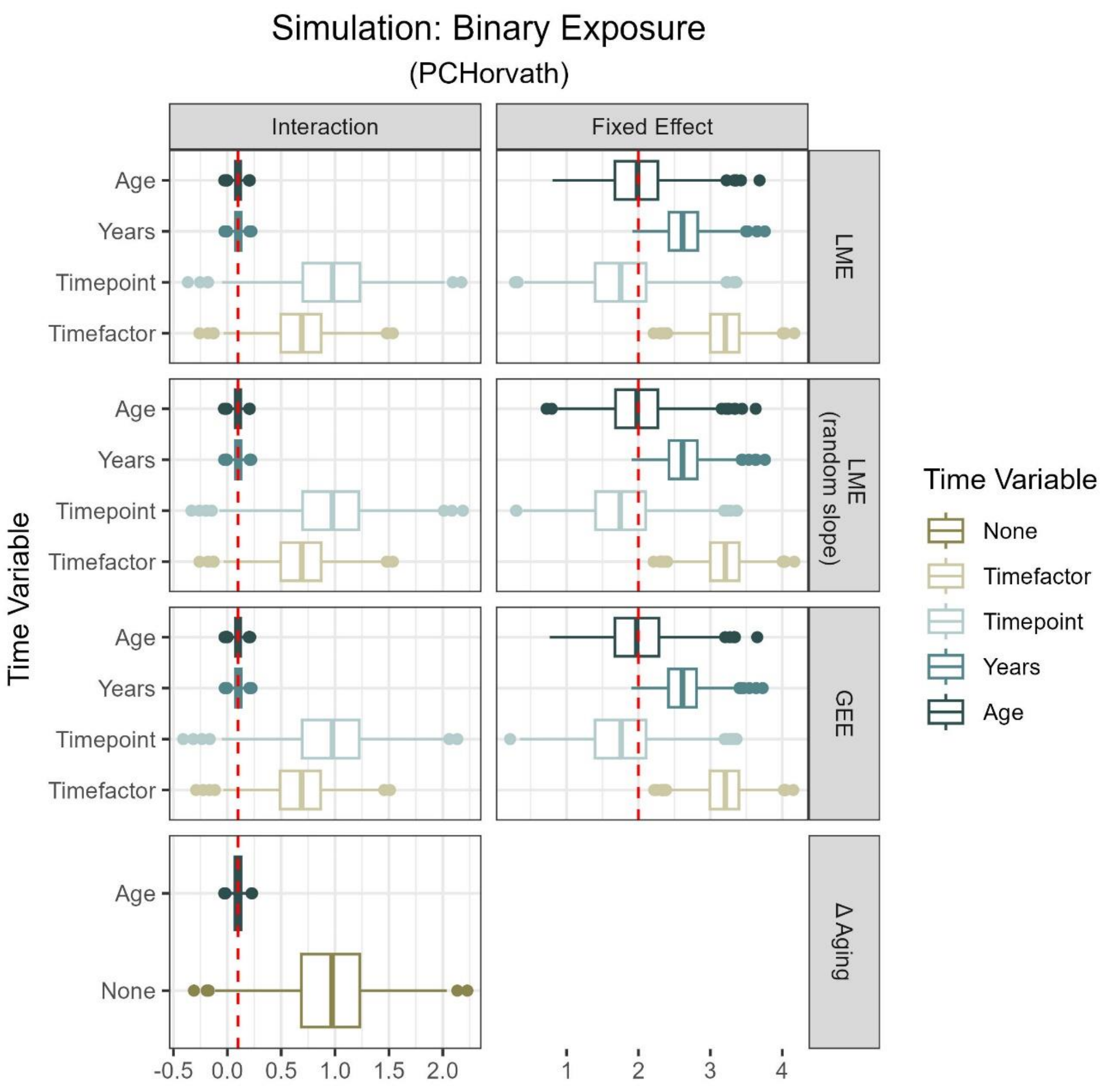

***Figure 5**: Binary exposure simulation results (n = 1,000) based on longitudinal ARIES data (two measurements, age 7 and 15-17)* [1]*. Rows show boxplots of effect size estimates derived from different models and time variables included in those models respectively. The two columns divide into interaction effect (left column) and fixed effect (right column). Simulated interaction effect of 0.1, and simulated fixed effect of 2, are marked in red. Models are Linear Mixed Effect Models (LME), Generalized Estimating Equations (GEE), and regression on difference between two epigenetic age (EA) measures (Δ aging). Time variables are chronological age (Age), years between measures (Years), number of measure (Timepoint, i.e., 1, 2, 3), factorized measure (Timefactor, i.e., F07, F09, F15). All models contained principal component Horvath clock* [4] *derived EA as outcome.*

1. Two Measure-based Simulation

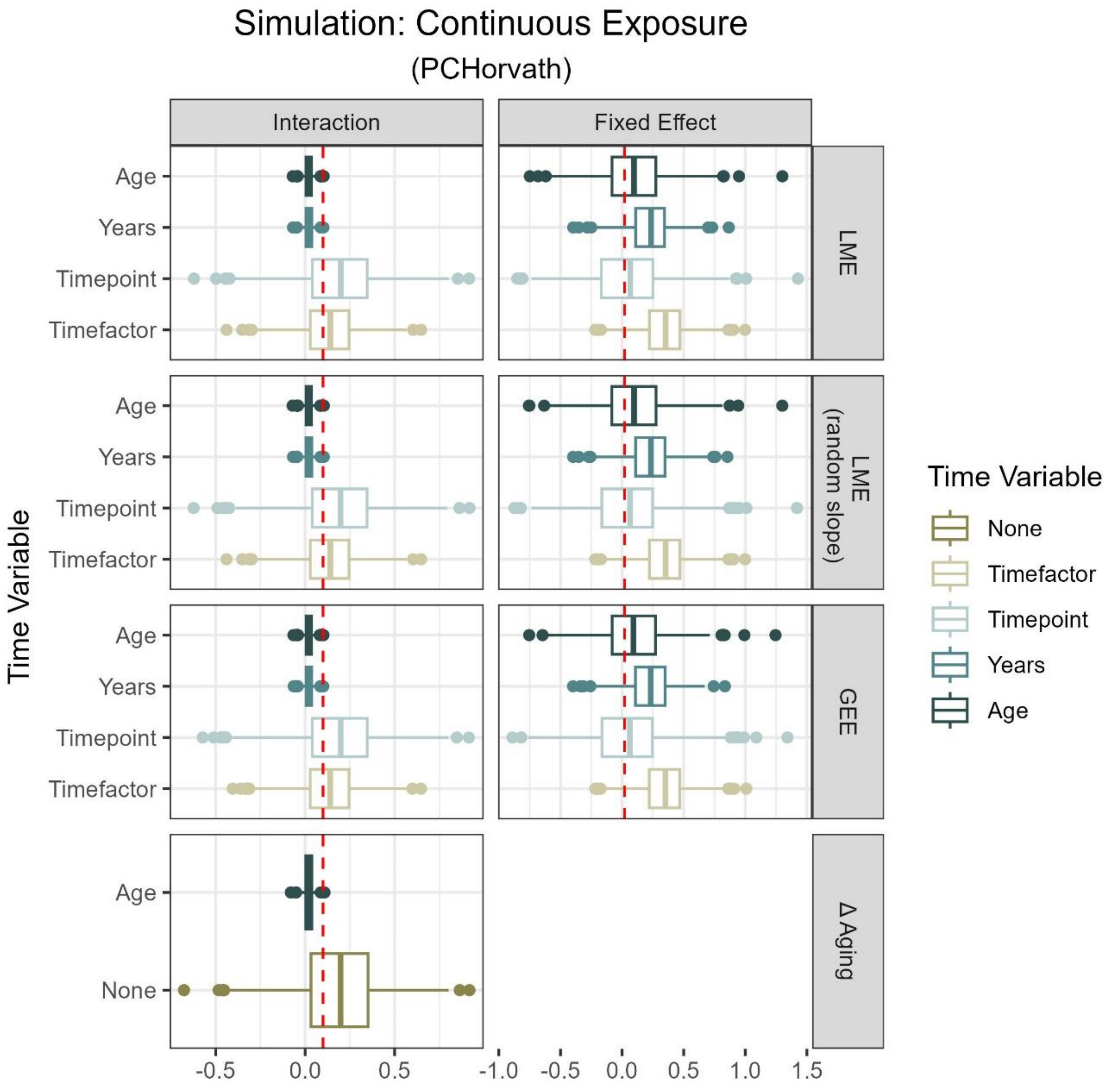

Figure 6*: Continuous exposure simulation results (n = 1,000) based on longitudinal ARIES data (two measurements, age 7 and 15-17)* [1]*. Rows show boxplots of effect size estimates derived from different models and time variables included in those models respectively. The two columns divide into interaction effect (left column) and fixed effect (right column). Simulated interaction effect of 0.02, and simulated fixed effect of 0.1, are marked in red. Models are Linear Mixed Effect Models (LME), Generalized Estimating Equations (GEE), and regression on difference between two epigenetic age (EA) measures (Δ aging). Time variables are chronological age (Age), years between measures (Years), number of measure (Timepoint, i.e., 1, 2, 3), factorized measure (Timefactor, i.e., F07, F09, F15). All models contained principal component Horvath clock* [4] *derived EA as outcome.*

1. Two Measure-based Simulation

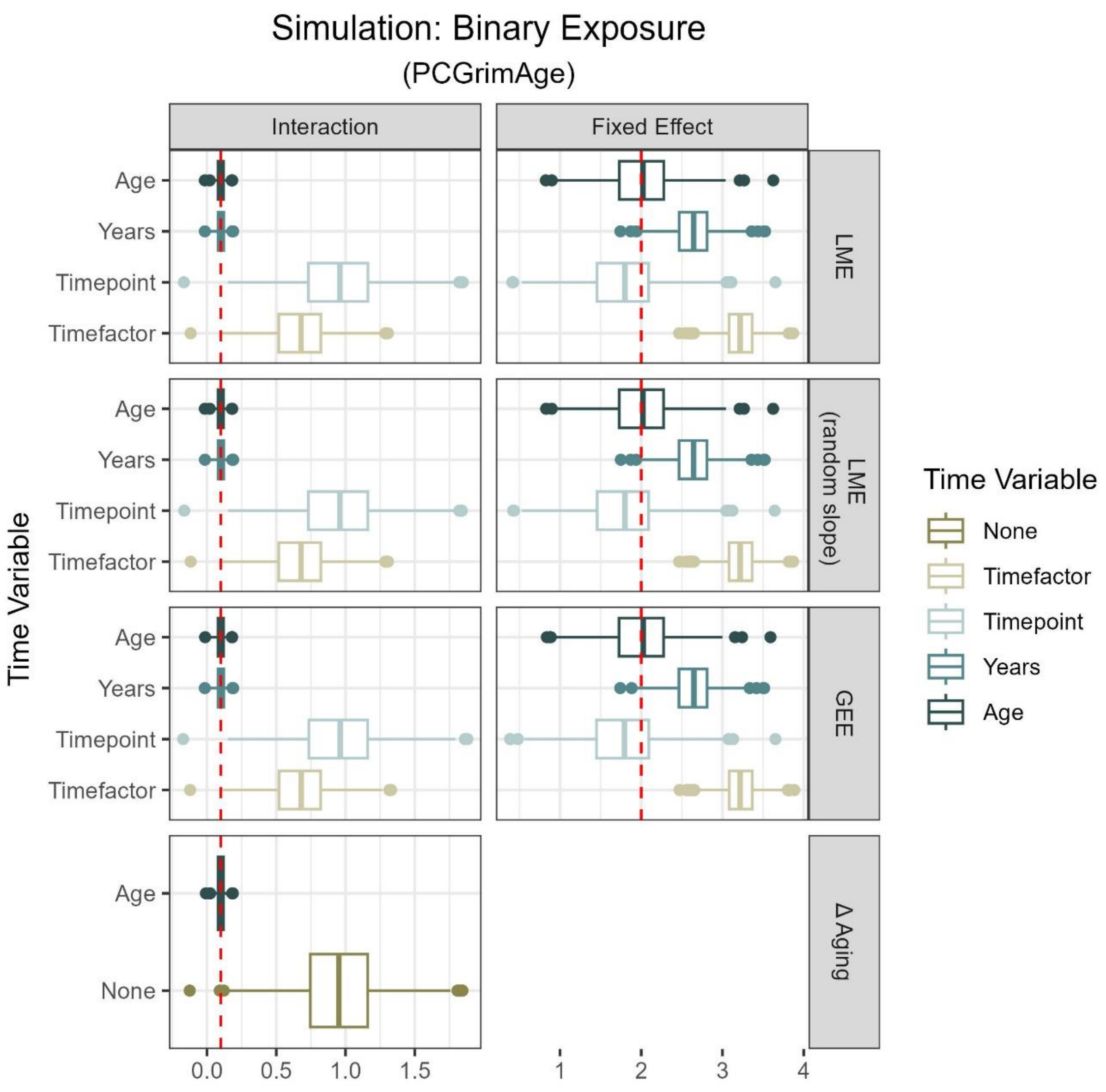

***Figure 7**: Binary exposure simulation results (n = 1,000) based on longitudinal ARIES data (two measurements, age 7 and 15-17)* [1]. *Rows show boxplots of effect size estimates derived from different models and time variables included in those models respectively. The two columns divide into interaction effect (left column) and fixed effect (right column). Simulated interaction effect of 0.1, and simulated fixed effect of 2, are marked in red. Models are Linear Mixed Effect Models (LME), Generalized Estimating Equations (GEE), and regression on difference between two epigenetic age (EA) measures (Δ aging). Time variables are chronological age (Age), years between measures (Years), number of measure (Timepoint, i.e., 1, 2, 3), factorized measure (Timefactor, i.e., F07, F09, F15). All models contained principal component GrimAge* [4] *derived EA as outcome.*

1. Two Measure-based Simulation

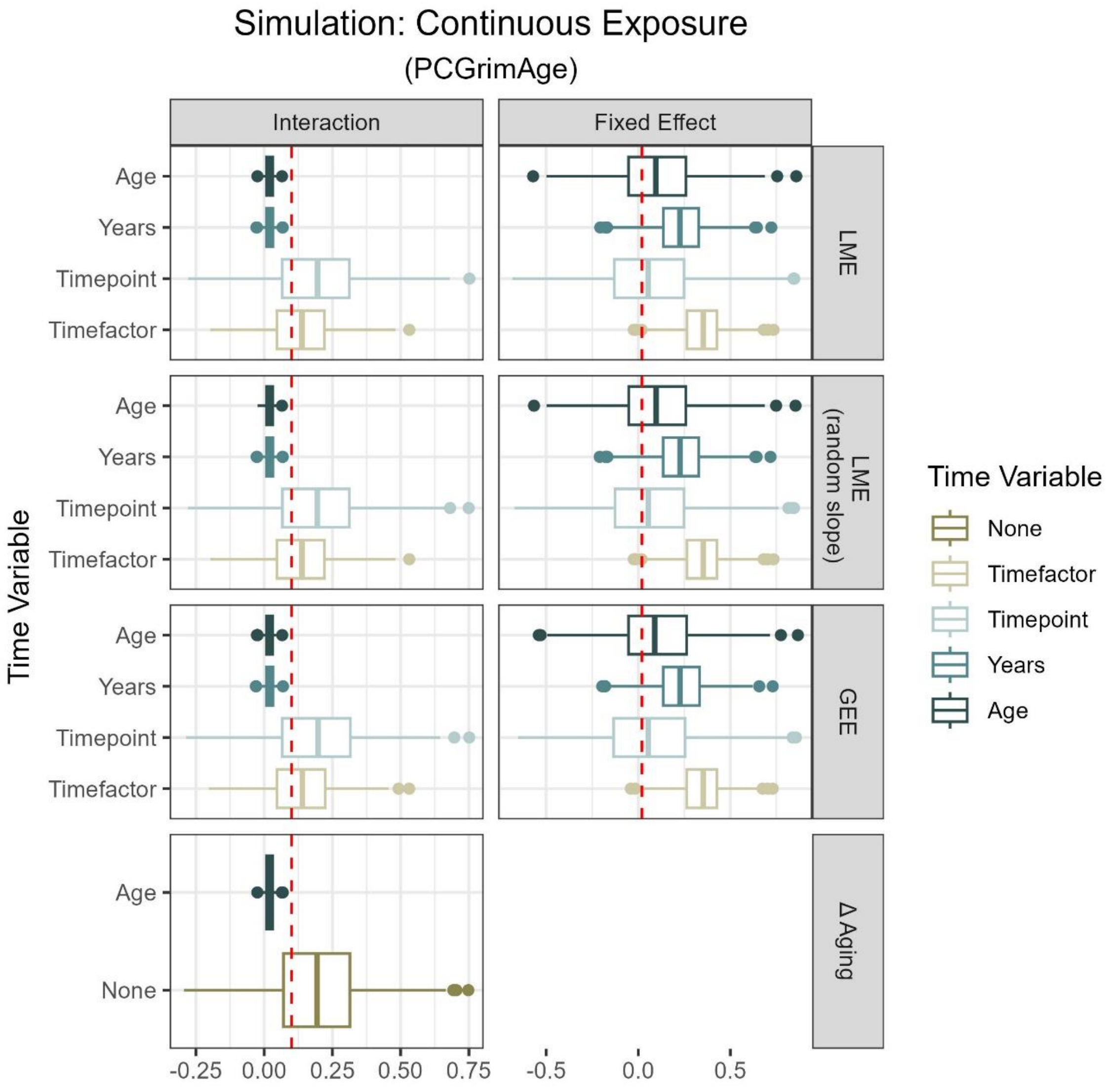

***Figure 8**: Continuous exposure simulation results (n = 1,000) based on longitudinal ARIES data (two measurements, age 7 and 15-17)* [1]*. Rows show boxplots of effect size estimates derived from different models and time variables included in those models respectively. The two columns divide into interaction effect (left column) and fixed effect (right column). Simulated interaction effect of 0.02, and simulated fixed effect of 0.1, are marked in red. Models are Linear Mixed Effect Models (LME), Generalized Estimating Equations (GEE), and regression on difference between two epigenetic age (EA) measures (Δ aging). Time variables are chronological age (Age), years between measures (Years), number of measure (Timepoint, i.e., 1, 2, 3), factorized measure (Timefactor, i.e., F07, F09, F15). All models contained principal component GrimAge* [4] *derived EA as outcome.*

1. Two Measure-based Simulation

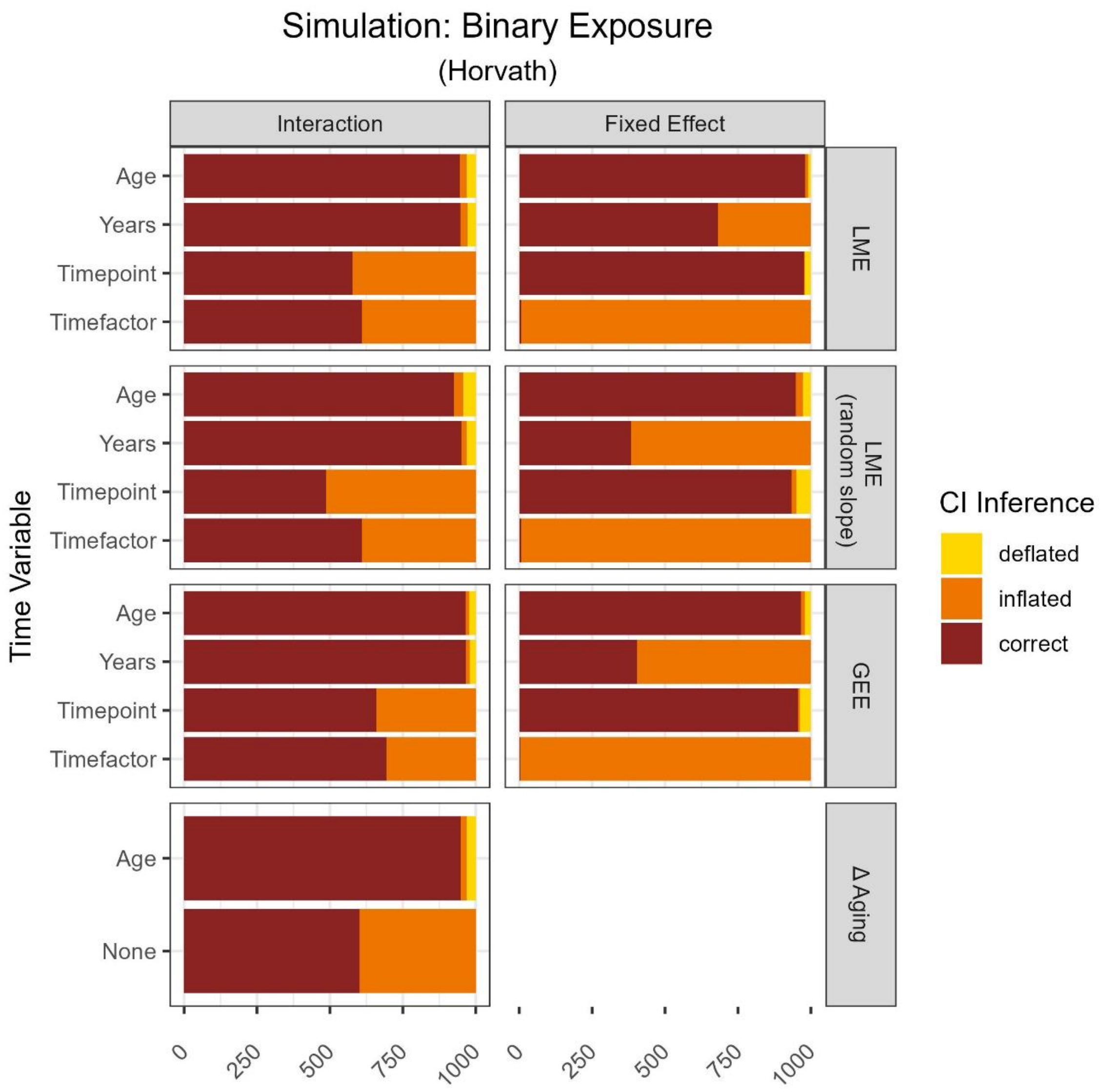

Figure 9*: Evaluation of confidence intervals (CI) from binary exposure simulation results (n = 1,000) based on longitudinal ARIES data (two measurements, age 7 and 15-17)* [1]*. Rows show the number of simulation results, from different models and time variables, falling in each of the three categories: inflated (CI fully above simulated effect size), deflated (CI fully below simulated effect size), and correct (CI contains simulated effect size). The two columns divide into interaction effect (left column) and fixed effect (right column). Models are Linear Mixed Effect Models (LME), Generalized Estimating Equations (GEE), and regression on difference between two epigenetic age (EA) measures (Δ aging). Time variables are chronological age (Age), years between measures (Years), number of measure (Timepoint, i.e., 1, 2, 3), factorized measure (Timefactor, i.e., F07, F09, F15). All models contained Horvath clock* [2] *derived EA as outcome.*

1. Two Measure-based Simulation

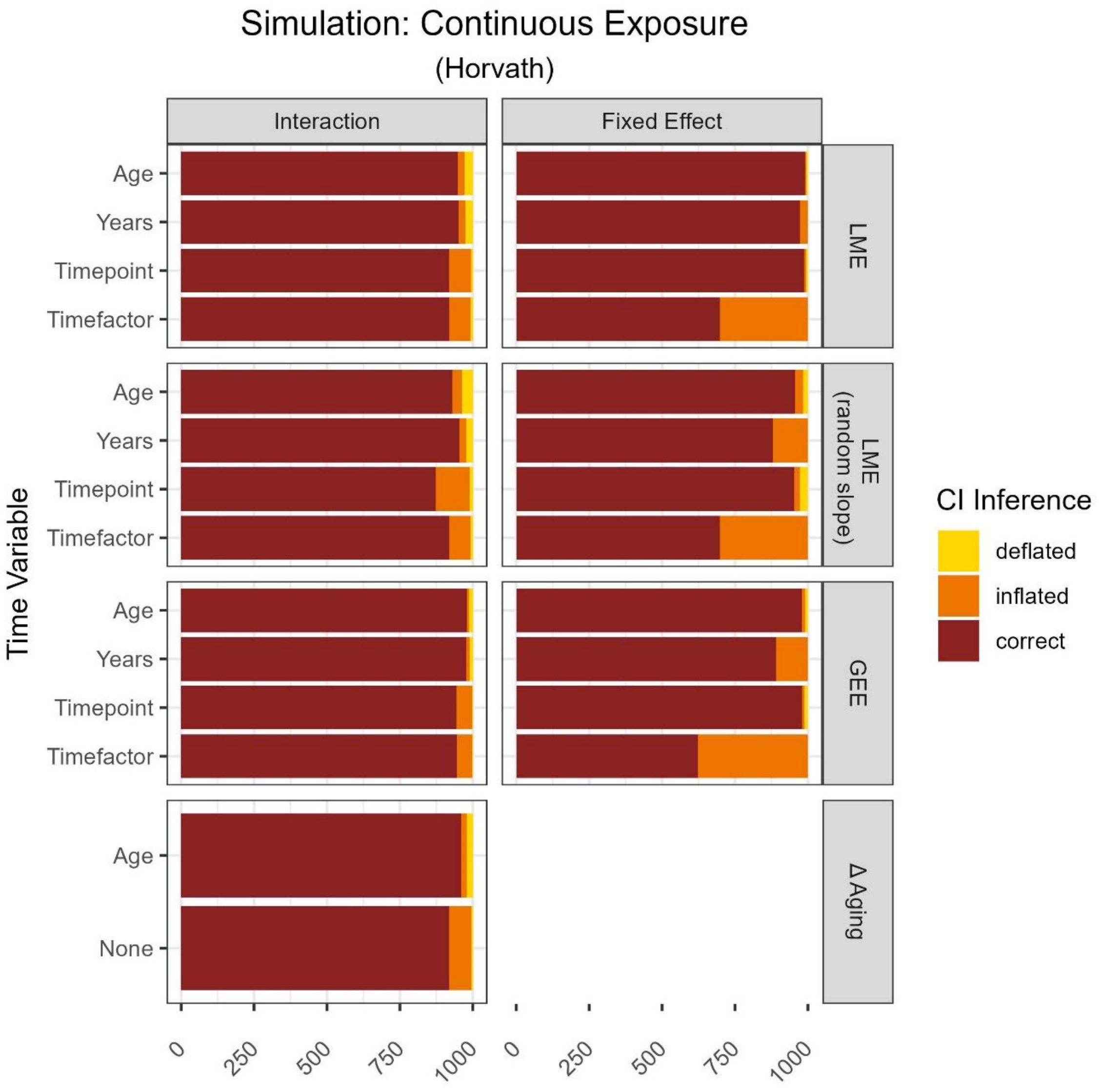

***Figure 10**: Evaluation of confidence intervals (CI) from continuous exposure simulation results (n = 1,000) based on longitudinal ARIES data (two measurements, age 7 and 15-17)* [1]. *Rows show the number of simulation results, from different models and time variables, falling in each of the three categories: inflated (CI fully above simulated effect size), deflated (CI fully below simulated effect size), and correct (CI contains simulated effect size). The two columns divide into interaction effect (left column) and fixed effect (right column). Models are Linear Mixed Effect Models (LME), Generalized Estimating Equations (GEE), and regression on difference between two epigenetic age (EA) measures (Δ aging). Time variables are chronological age (Age), years between measures (Years), number of measure (Timepoint, i.e., 1, 2, 3), factorized measure (Timefactor, i.e., F07, F09, F15). All models contained Horvath clock* [2] *derived EA as outcome.*

1. Two Measure-based Simulation

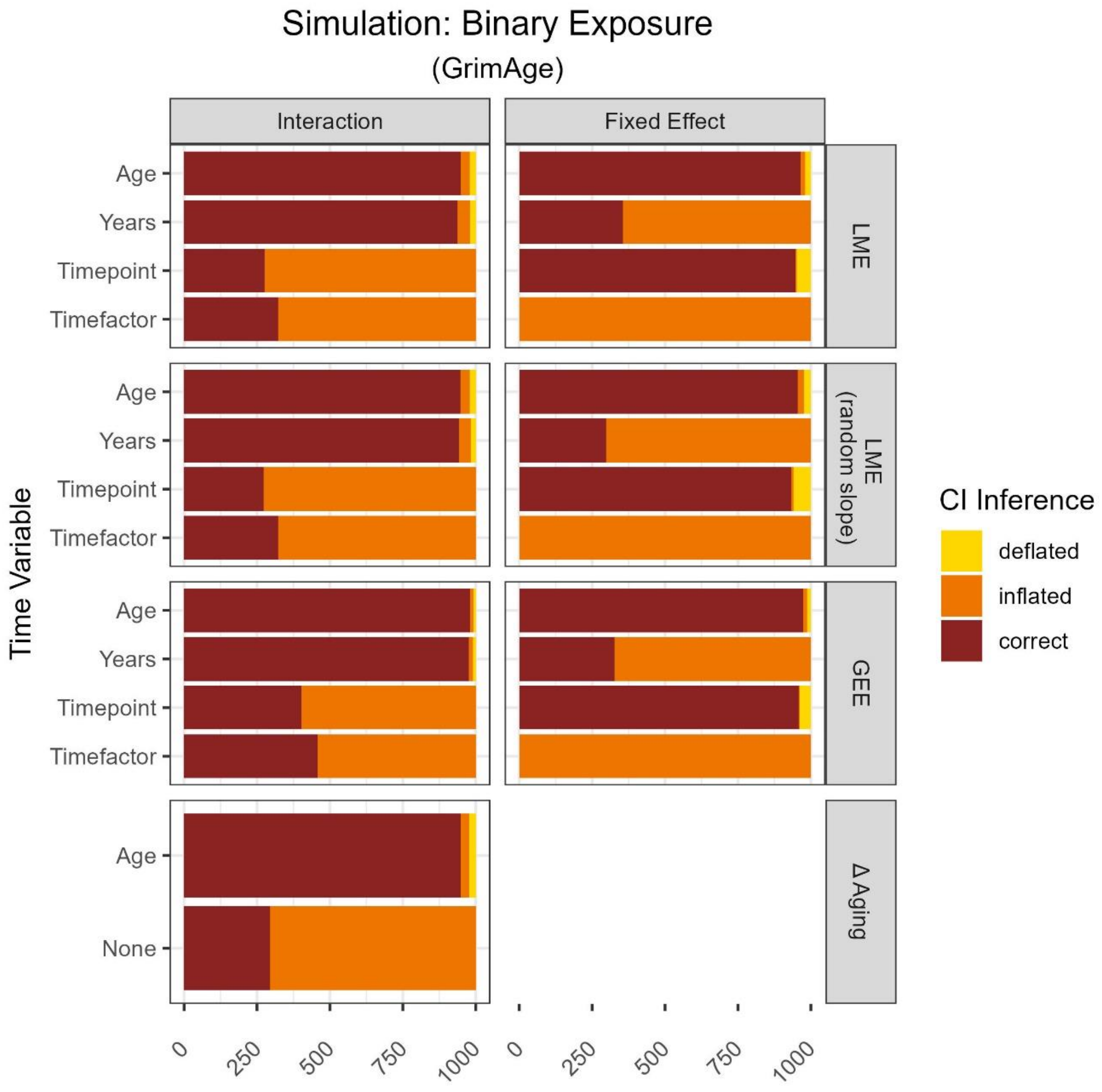

***Figure 11**: Evaluation of confidence intervals (CI) from binary exposure simulation results (n = 1,000) based on longitudinal ARIES data (two measurements, age 7 and 15-17)* [1]. *Rows show the number of simulation results, from different models and time variables, falling in each of the three categories: inflated (CI fully above simulated effect size), deflated (CI fully below simulated effect size), and correct (CI contains simulated effect size). The two columns divide into interaction effect (left column) and fixed effect (right column). Models are Linear Mixed Effect Models (LME), Generalized Estimating Equations (GEE), and regression on difference between two epigenetic age (EA) measures (Δ aging). Time variables are chronological age (Age), years between measures (Years), number of measure (Timepoint, i.e., 1, 2, 3), factorized measure (Timefactor, i.e., F07, F09, F15). All models contained GrimAge* [3] *derived EA as outcome.*

1. Two Measure-based Simulation

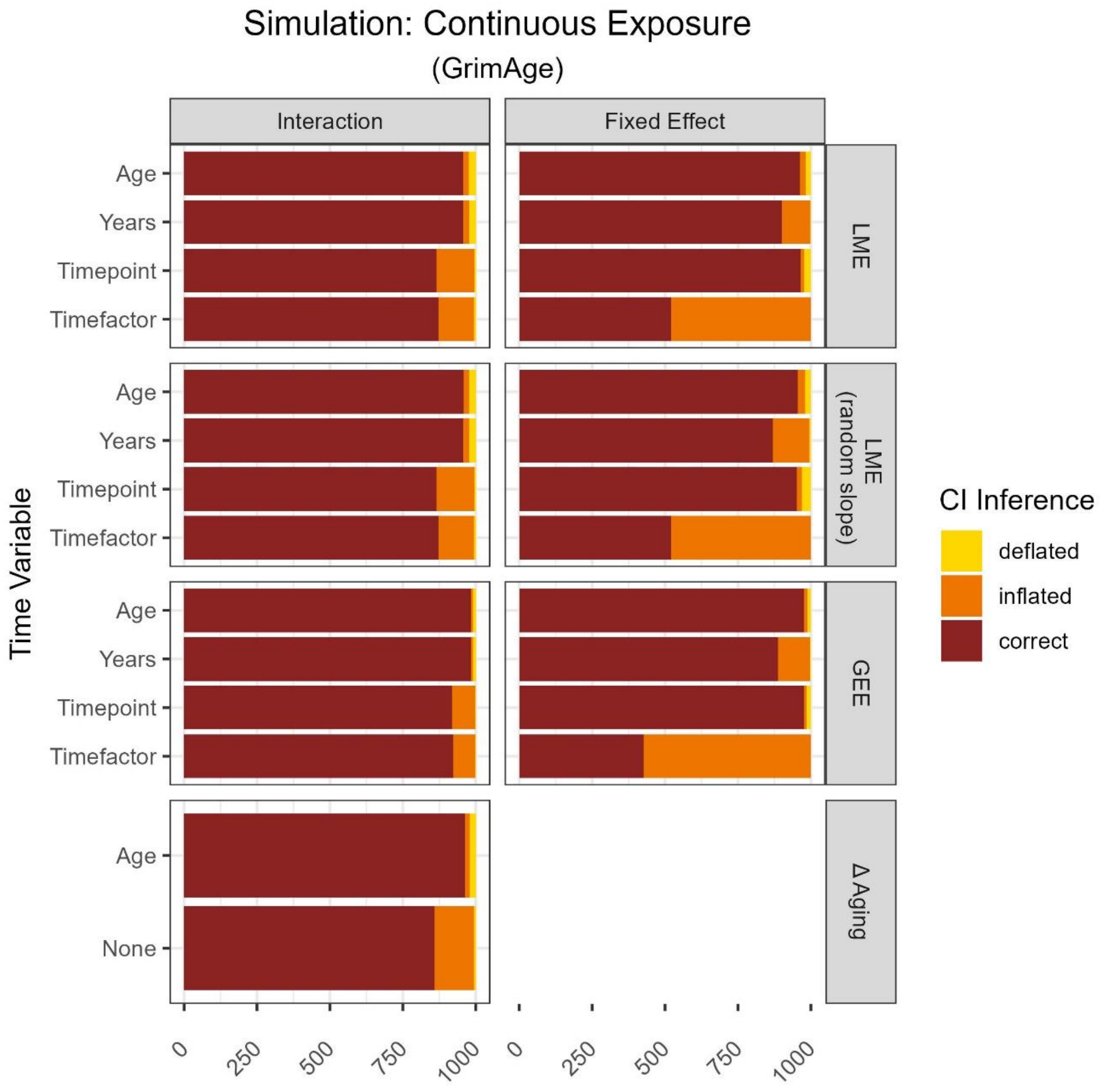

***Figure 12**: Evaluation of confidence intervals (CI) from continuous exposure simulation results (n = 1,000) based on longitudinal ARIES data (two measurements, age 7 and 15-17)* [1]. *Rows show the number of simulation results, from different models and time variables, falling in each of the three categories: inflated (CI fully above simulated effect size), deflated (CI fully below simulated effect size), and correct (CI contains simulated effect size). The two columns divide into interaction effect (left column) and fixed effect (right column). Models are Linear Mixed Effect Models (LME), Generalized Estimating Equations (GEE), and regression on difference between two epigenetic age (EA) measures (Δ aging). Time variables are chronological age (Age), years between measures (Years), number of measure (Timepoint, i.e., 1, 2, 3), factorized measure (Timefactor, i.e., F07, F09, F15). All models contained GrimAge* [3] *derived EA as outcome.*

1. Two Measure-based Simulation

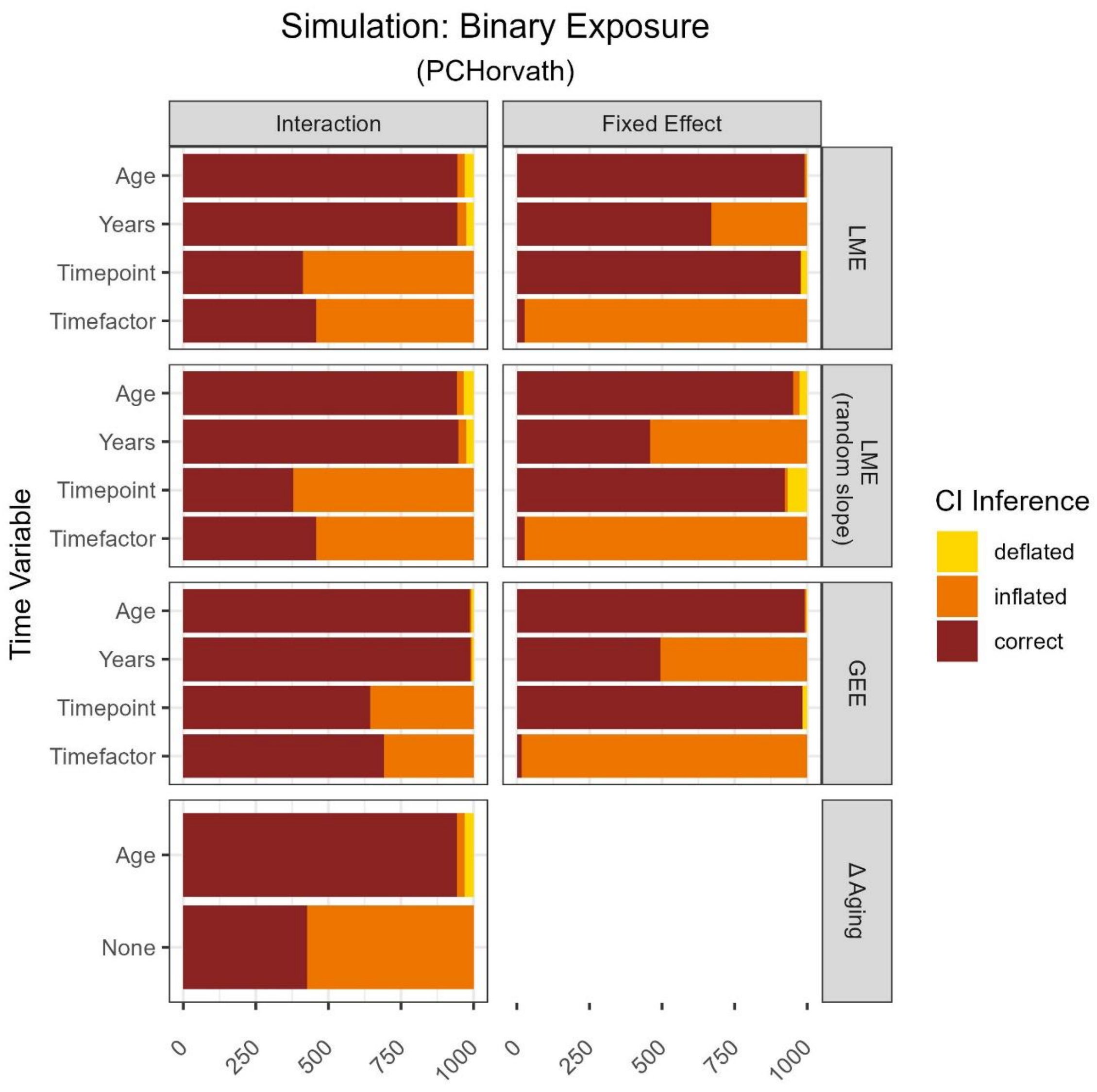

Figure 13: *Evaluation of confidence intervals (CI) from binary exposure simulation results (n = 1,000) based on longitudinal ARIES data (two measurements, age 7 and 15-17)* [1]. *Rows show the number of simulation results, from different models and time variables, falling in each of the three categories: inflated (CI fully above simulated effect size), deflated (CI fully below simulated effect size), and correct (CI contains simulated effect size). The two columns divide into interaction effect (left column) and fixed effect (right column). Models are Linear Mixed Effect Models (LME), Generalized Estimating Equations (GEE), and regression on difference between two epigenetic age (EA) measures (Δ aging). Time variables are chronological age (Age), years between measures (Years), number of measure (Timepoint, i.e., 1, 2, 3), factorized measure (Timefactor, i.e., F07, F09, F15). All models contained principal component Horvath clock* [4] *derived EA as outcome.*

1. Two Measure-based Simulation

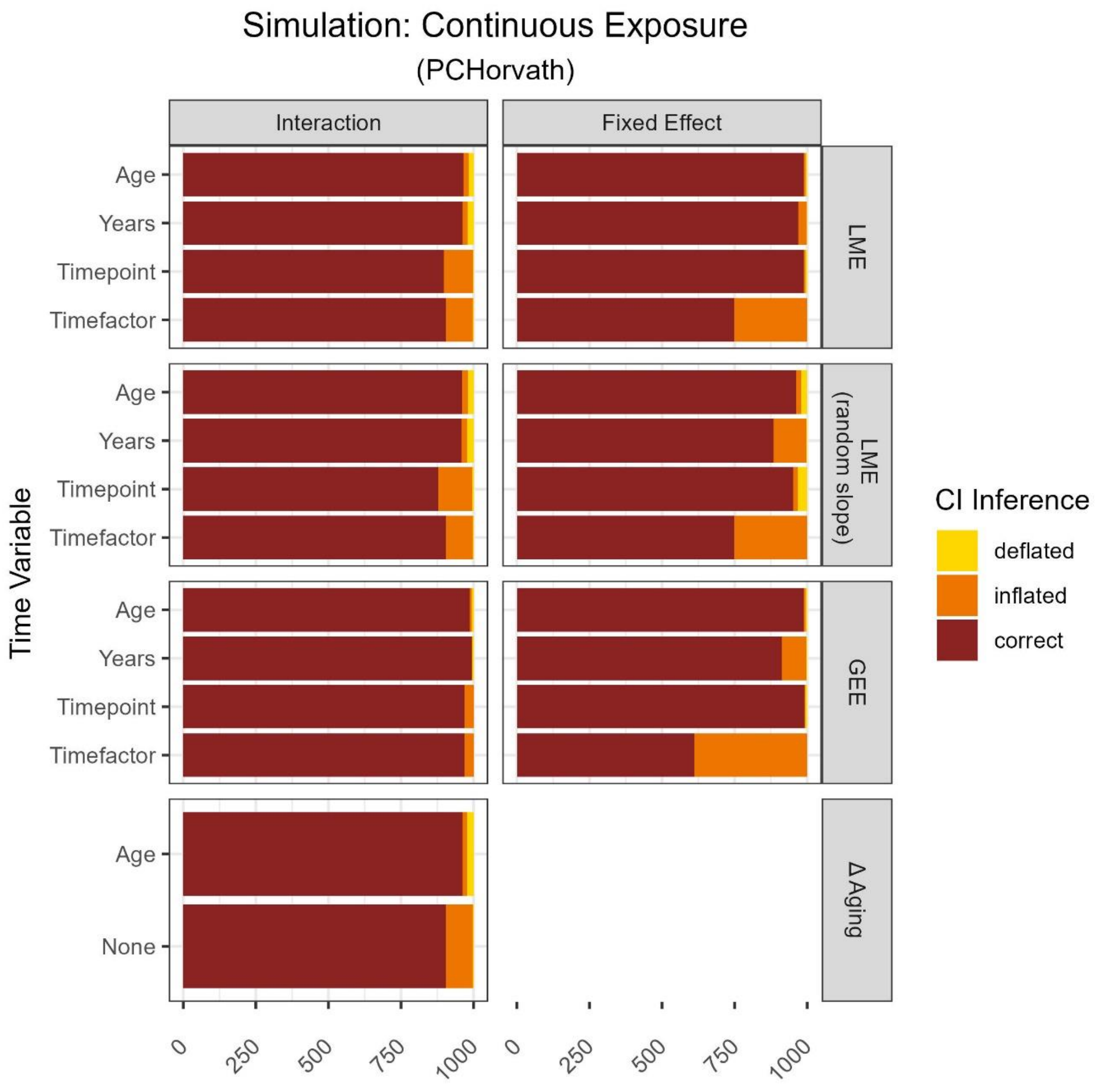

***Figure 14**: Evaluation of confidence intervals (CI) from continuous exposure simulation results (n = 1,000) based on longitudinal ARIES data (two measurements, age 7 and 15-17)* [1]. *Rows show the number of simulation results, from different models and time variables, falling in each of the three categories: inflated (CI fully above simulated effect size), deflated (CI fully below simulated effect size), and correct (CI contains simulated effect size). The two columns divide into interaction effect (left column) and fixed effect (right column). Models are Linear Mixed Effect Models (LME), Generalized Estimating Equations (GEE), and regression on difference between two epigenetic age (EA) measures (Δ aging). Time variables are chronological age (Age), years between measures (Years), number of measure (Timepoint, i.e., 1, 2, 3), factorized measure (Timefactor, i.e., F07, F09, F15). All models contained principal component Horvath clock* [4] *derived EA as outcome.*

1. Two Measure-based Simulation

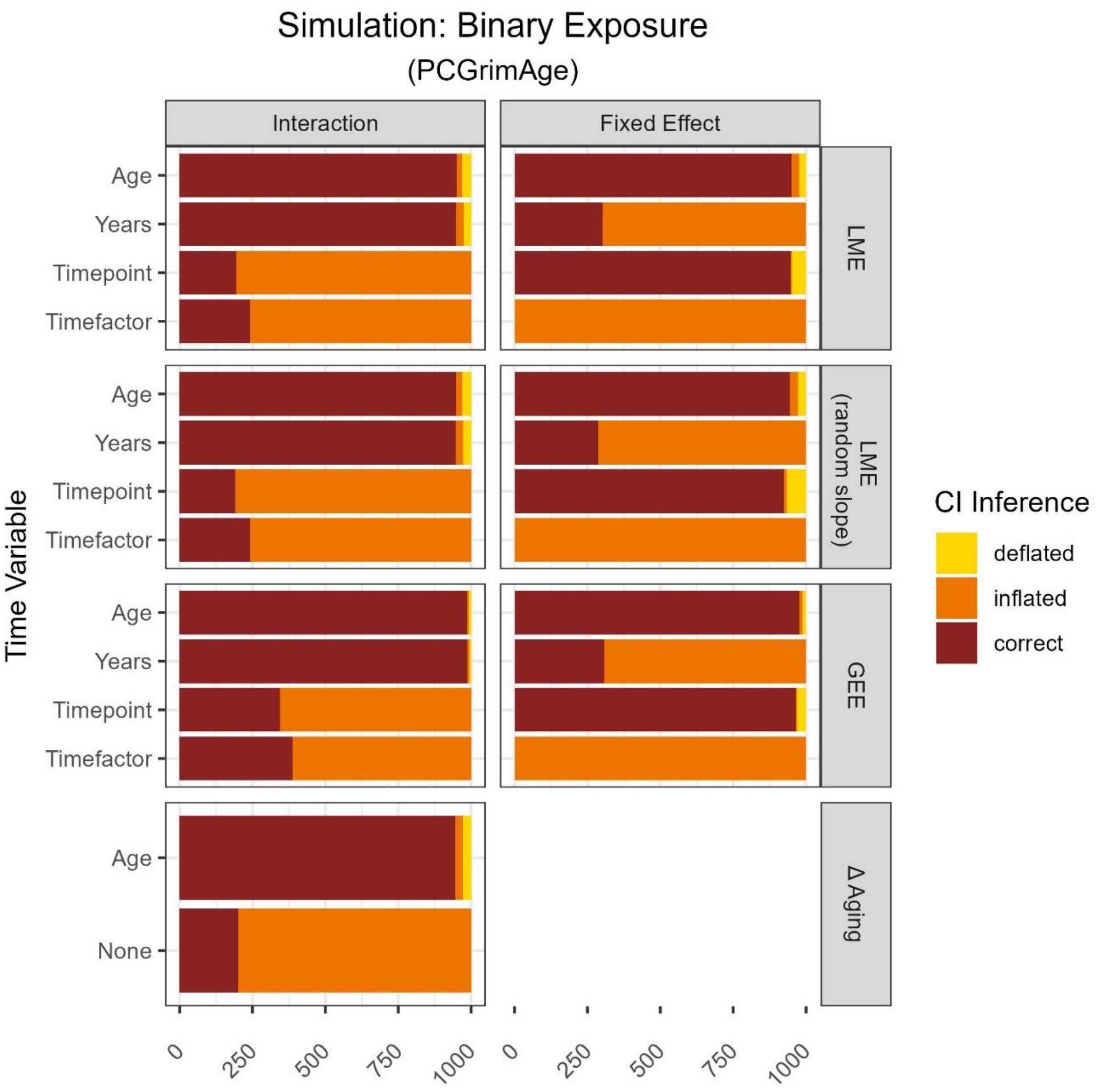

***Figure 15**: Evaluation of confidence intervals (CI) from binary exposure simulation results (n = 1,000) based on longitudinal ARIES data (two measurements, age 7 and 15-17)* [1]. *Rows show the number of simulation results, from different models and time variables, falling in each of the three categories: inflated (CI fully above simulated effect size), deflated (CI fully below simulated effect size), and correct (CI contains simulated effect size). The two columns divide into interaction effect (left column) and fixed effect (right column). Models are Linear Mixed Effect Models (LME), Generalized Estimating Equations (GEE), and regression on difference between two epigenetic age (EA) measures (Δ aging). Time variables are chronological age (Age), years between measures (Years), number of measure (Timepoint, i.e., 1, 2, 3), factorized measure (Timefactor, i.e., F07, F09, F15). All models contained principal component GrimAge* [4] *derived EA as outcome.*

1. Two Measure-based Simulation

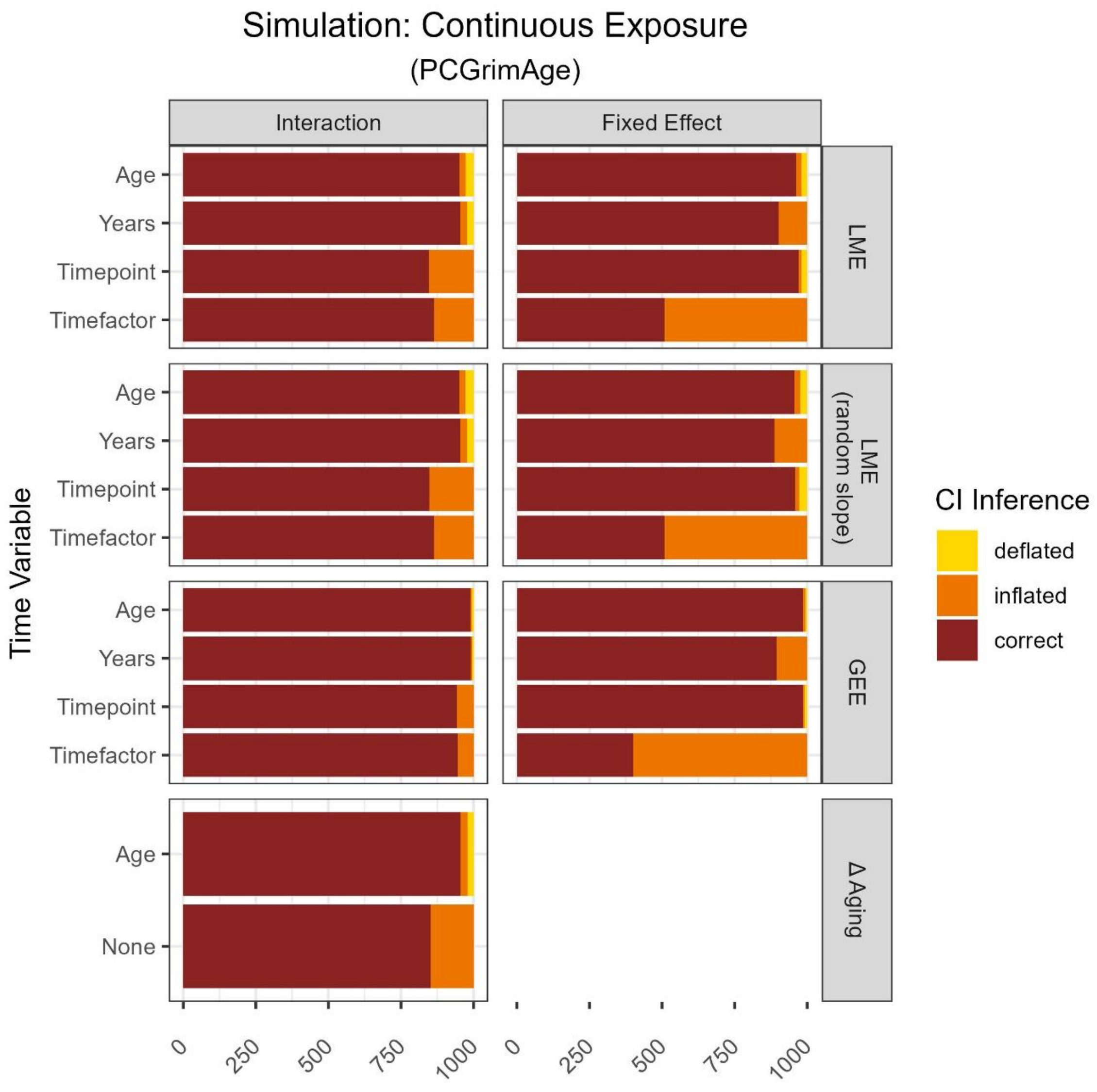

***Figure 16**: Evaluation of confidence intervals (CI) from continuous exposure simulation results (n = 1,000) based on longitudinal ARIES data (two measurements, age 7 and 15-17)* [1]*. Rows show the number of simulation results, from different models and time variables, falling in each of the three categories: inflated (CI fully above simulated effect size), deflated (CI fully below simulated effect size), and correct (CI contains simulated effect size). The two columns divide into interaction effect (left column) and fixed effect (right column). Models are Linear Mixed Effect Models (LME), Generalized Estimating Equations (GEE), and regression on difference between two epigenetic age (EA) measures (Δ aging). Time variables are chronological age (Age), years between measures (Years), number of measure (Timepoint, i.e., 1, 2, 3), factorized measure (Timefactor, i.e., F07, F09, F15). All models contained principal component GrimAge* [4] *derived EA as outcome.*

2. Three Measure-based Simulation

***Table 5**: Summary of fixed effect estimates and confidence intervals (CI) from simulations (n = 1,000) based on ARIES cohort data (three measurements, age 7, 9, and 15-17)* [1]. *Binary (effect size: 2) as well as continuous exposure (effect size: 0.1) were simulated separately. Methods used to model the simulated exposures differed in (i) the type of model, (ii) the outcome and (iii) time variables included in the model. Rows show results from each model and time variable. Columns divide results in outcome variables included in the model. Models are Linear Mixed Effect Models (LME) and Generalized Estimating Equations (GEE). Time variables are chronological age (Age), years between measures (Years), number of measure (Timepoint, i.e., 1, 2, 3), factorized measure (Timefactor, i.e., F07, F09, F15). Outcome variables are EAA (i.e., residual from regressing EA on age), or EA itself, derived from either the Horvath clock* [2] *or GrimAge* [3]. *We calculated the CI as mean estimate ± mean standard error across all simulations.*

**Model**	**Time Variable**	**Outcome**							
		Horvath		GrimAge		Horvath		GrimAge	
		EA	EAA	EA	EAA	EA	EAA	EA	EAA
		**Fixed Effect** Estimate (CI)		**Fixed Effect** Estimate (CI)		**Fixed Effect** Estimate (CI)		**Fixed Effect** Estimate (CI)	
LME	Age	2 (0.55,3.44)	1.99 (0.57,3.4)	1.97 (0.81,3.14)	1.96 (0.86,3.07)	0.08 (-0.72,0.89)	0.08 (-0.71,0.88)	0.1 (-0.55,0.75)	0.1 (-0.52,0.72)
	Years	2.76 (2.01,3.51)	2.75 (2,3.5)	2.73 (2.11,3.36)	2.73 (2.12,3.33)	0.24 (-0.18,0.66)	0.24 (-0.18,0.66)	0.25 (-0.1,0.6)	0.25 (-0.09,0.59)
	Timepoint	2.22 (0.86,3.59)	2.21 (0.99,3.44)	2.2 (1.04,3.36)	2.19 (1.23,3.15)	0.13 (-0.64,0.91)	0.13 (-0.55,0.82)	0.15 (-0.51,0.81)	0.15 (-0.39,0.68)
	Timefactor	8.81 (8.48,9.13)	-0.07 (-0.39,0.24)	8.31 (8.05,8.57)	-0.05 (-0.3,0.19)	8.78 (6.59,10.98)	-0.69 (-2.83,1.45)	8.33 (6.57,10.08)	-0.63 (-2.28,1.01)
LME (random slope)	Age	2 (0.73,3.27)	1.99 (0.75,3.22)	1.97 (0.81,3.13)	1.96 (0.87,3.06)	0.09 (-0.63,0.8)	0.08 (-0.61,0.78)	0.1 (-0.55,0.75)	0.1 (-0.51,0.71)
	Years	2.76 (2.13,3.39)	2.75 (2.13,3.37)	2.73 (2.11,3.35)	2.73 (2.12,3.33)	0.24 (-0.11,0.6)	0.24 (-0.11,0.59)	0.25 (-0.1,0.6)	0.25 (-0.09,0.59)
	Timepoint	2.23 (1.04,3.42)	2.22 (1.19,3.26)	2.2 (1,3.4)	2.19 (1.24,3.14)	0.13 (-0.54,0.81)	0.13 (-0.45,0.71)	0.15 (-0.53,0.83)	0.15 (-0.39,0.68)
	Timefactor	8.8 (8.47,9.14)	-0.07 (-0.4,0.25)	8.31 (8.02,8.59)	-0.06 (-0.34,0.22)	8.78 (6.5,11.06)	-0.7 (-2.89,1.5)	8.32 (6.37,10.28)	-0.64 (-2.52,1.24)
GEE	Age	2 (0.62,3.38)	1.99 (0.63,3.35)	1.97 (0.76,3.19)	1.96 (0.78,3.15)	0.08 (-0.7,0.86)	0.08 (-0.69,0.86)	0.1 (-0.58,0.78)	0.1 (-0.57,0.76)
	Years	2.76 (2.15,3.37)	2.75 (2.15,3.35)	2.73 (2.14,3.33)	2.73 (2.14,3.31)	0.24 (-0.1,0.59)	0.24 (-0.1,0.58)	0.25 (-0.09,0.59)	0.25 (-0.08,0.58)
	Timepoint	2.23 (1.08,3.37)	2.22 (1.12,3.32)	2.2 (1.15,3.26)	2.19 (1.2,3.19)	0.13 (-0.52,0.78)	0.13 (-0.49,0.76)	0.15 (-0.45,0.74)	0.15 (-0.41,0.71)
	Timefactor	8.81 (8.45,9.16)	-0.07 (-0.42,0.27)	8.31 (8.02,8.59)	-0.06 (-0.33,0.21)	8.78 (6.37,11.19)	-0.7 (-3.06,1.66)	8.32 (6.4,10.25)	-0.64 (-2.47,1.19)
**Exposure**		Binary Simulated Fixed Effect: **2.0**				Continuous Simulated Fixed Effect: **0.1**			

2. Three Measure-based Simulation

Table 6*: Summary of interaction effect estimates and confidence intervals (CI) from simulations (n = 1,000) based on ARIES cohort data (three measurements, age 7, 9, and 15-17)* [1]*. Binary (effect size: 0.1) as well as continuous exposure (effect size: 0.02) were simulated separately. Methods used to model the simulated exposures differed in (i) the type of model, (ii) the outcome and (iii) time variables included in the model. Rows show results from each model and time variable. Columns divide results in outcome variables included in the model. Models are Linear Mixed Effect Models (LME) and Generalized Estimating Equations (GEE). Time variables are chronological age (Age), years between measures (Years), number of measure (Timepoint, i.e., 1, 2, 3), factorized measure (Timefactor, i.e., F07, F09, F15). Outcome variables are EAA (i.e., residual from regressing EA on age), or EA itself, derived from either the Horvath clock* [2] *or GrimAge* [3]*. We calculated the CI as mean estimate ± mean standard error across all simulations.*

**Model**	**Time Variable**	**Outcome**							
		Horvath		GrimAge		Horvath		GrimAge	
		EA	EAA	EA	EAA	EA	EAA	EA	EAA
		**Interaction** Estimate (CI)		**Interaction** Estimate (CI)		**Interaction** Estimate (CI)		**Interaction** Estimate (CI)	
LME	Age	0.1 (-0.01,0.21)	0.1 (-0.01,0.21)	0.1 (0.01,0.19)	0.1 (0.02,0.18)	0.02 (-0.04,0.08)	0.02 (-0.04,0.08)	0.02 (-0.03,0.07)	0.02 (-0.03,0.07)
	Years	0.09 (-0.01,0.19)	0.09 (-0.01,0.19)	0.09 (0.01,0.17)	0.09 (0.02,0.17)	0.02 (-0.04,0.08)	0.02 (-0.04,0.08)	0.02 (-0.03,0.06)	0.02 (-0.02,0.06)
	Timepoint	0.48 (-0.13,1.09)	0.49 (-0.05,1.03)	0.48 (-0.04,1)	0.49 (0.07,0.9)	0.1 (-0.25,0.45)	0.1 (-0.2,0.4)	0.09 (-0.2,0.39)	0.09 (-0.14,0.33)
	Timefactor	-0.72 (-1.05,-0.39)	-0.71 (-1.04,-0.39)	-1.87 (-2.15,-1.59)	-1.85 (-2.12,-1.58)	-0.72 (-1.05,-0.39)	-0.71 (-1.04,-0.39)	-1.87 (-2.15,-1.59)	-1.85 (-2.13,-1.58)
LME (random slope)	Age	0.1 (-0.01,0.21)	0.1 (0,0.21)	0.1 (0.01,0.19)	0.1 (0.02,0.19)	0.02 (-0.04,0.08)	0.02 (-0.04,0.08)	0.02 (-0.03,0.07)	0.02 (-0.03,0.07)
	Years	0.09 (-0.02,0.2)	0.09 (-0.01,0.2)	0.09 (0.01,0.17)	0.09 (0.02,0.17)	0.02 (-0.04,0.08)	0.02 (-0.04,0.08)	0.02 (-0.03,0.06)	0.02 (-0.02,0.06)
	Timepoint	0.48 (-0.1,1.06)	0.48 (-0.04,1)	0.48 (-0.06,1.03)	0.48 (0.07,0.9)	0.1 (-0.23,0.43)	0.1 (-0.19,0.39)	0.09 (-0.22,0.4)	0.09 (-0.14,0.33)
	Timefactor	-0.6 (-0.91,-0.3)	-0.58 (-0.89,-0.28)	-1.85 (-2.11,-1.6)	-1.83 (-2.07,-1.58)	-0.61 (-0.91,-0.3)	-0.58 (-0.89,-0.28)	-1.85 (-2.11,-1.6)	-1.83 (-2.07,-1.58)
GEE	Age	0.1 (-0.02,0.22)	0.1 (-0.02,0.22)	0.1 (0.01,0.2)	0.1 (0.01,0.19)	0.02 (-0.05,0.09)	0.02 (-0.05,0.09)	0.02 (-0.03,0.07)	0.02 (-0.03,0.07)
	Years	0.09 (-0.02,0.2)	0.09 (-0.02,0.2)	0.09 (0,0.18)	0.09 (0.01,0.18)	0.02 (-0.05,0.08)	0.02 (-0.04,0.08)	0.02 (-0.03,0.07)	0.02 (-0.03,0.07)
	Timepoint	0.48 (-0.13,1.09)	0.49 (-0.1,1.07)	0.48 (-0.01,0.98)	0.49 (0.03,0.94)	0.1 (-0.25,0.45)	0.1 (-0.23,0.43)	0.09 (-0.18,0.37)	0.09 (-0.16,0.35)
	Timefactor	-0.71 (-1.02,-0.4)	-0.7 (-1,-0.4)	-1.85 (-2.11,-1.6)	-1.83 (-2.07,-1.58)	-0.71 (-1.02,-0.4)	-0.7 (-1,-0.4)	-1.85 (-2.11,-1.6)	-1.83 (-2.07,-1.58)
**Exposure**		Binary Simulated Interaction Effect: **0.1**				Continuous Simulated Interaction Effect: **0.02**			

2. Three Measure-based Simulation

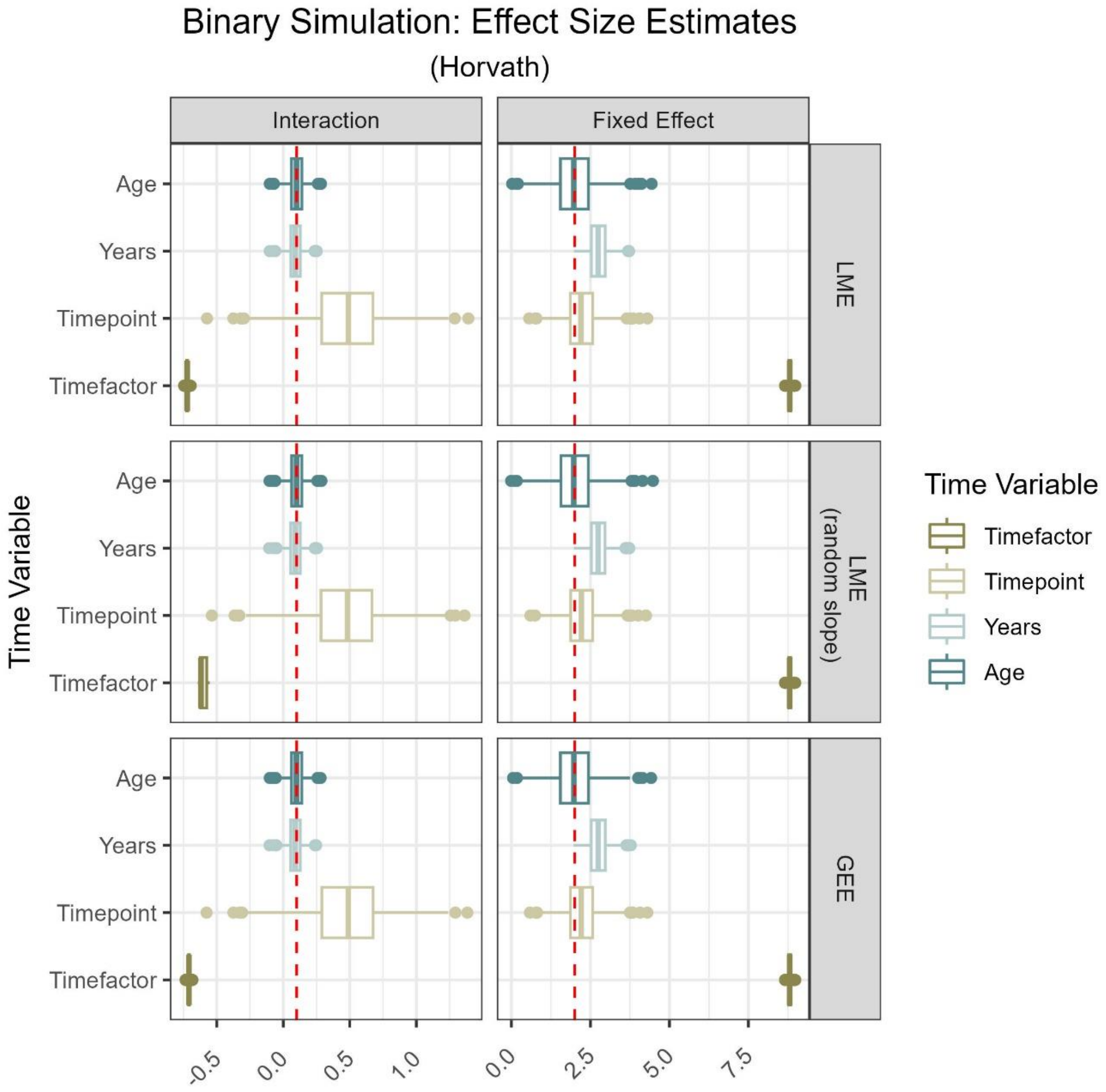

***Figure 17**: Binary exposure simulation results (n = 1,000) based on longitudinal ARIES data data (three measurements, age 7, 9, and 15-17)* [1]*. Rows show boxplots of effect size estimates derived from different models and time variables included in those models respectively. The two columns divide into interaction effect (left column) and fixed effect (right column). Simulated interaction effect of 0.1, and simulated fixed effect of 2, are marked in red. Models are Linear Mixed Effect Models (LME) and Generalized Estimating Equations (GEE). Time variables are chronological age (Age), years between measures (Years), number of measure (Timepoint, i.e., 1, 2, 3), factorized measure (Timefactor, i.e., F07, F09, F15). All models contained Horvath clock* [2] *derived EA as outcome.*

2. Three Measure-based Simulation

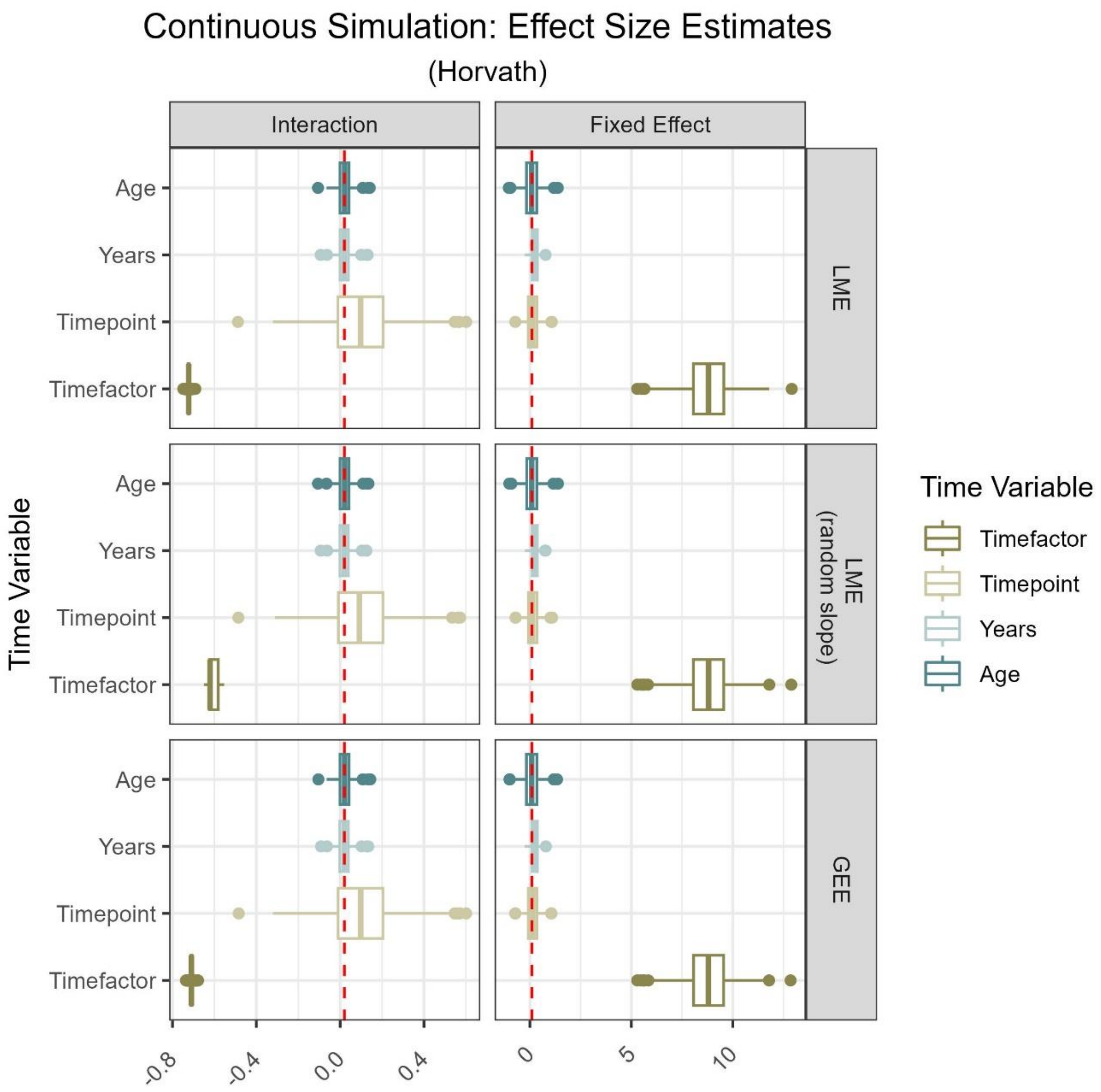

***Figure 18**: Continuous exposure simulation results (n = 1,000) based on longitudinal ARIES data (three measurements, age 7, 9, and 15-17)* [1]*. Rows show boxplots of effect size estimates derived from different models and time variables included in those models respectively. The two columns divide into interaction effect (left column) and fixed effect (right column). Simulated interaction effect of 0.02, and simulated fixed effect of 0.1, are marked in red. Models are Linear Mixed Effect Models (LME) and Generalized Estimating Equations (GEE). Time variables are chronological age (Age), years between measures (Years), number of measure (Timepoint, i.e., 1, 2, 3), factorized measure (Timefactor, i.e., F07, F09, F15). All models contained Horvath clock* [2] *derived EA as outcome.*

2. Three Measure-based Simulation

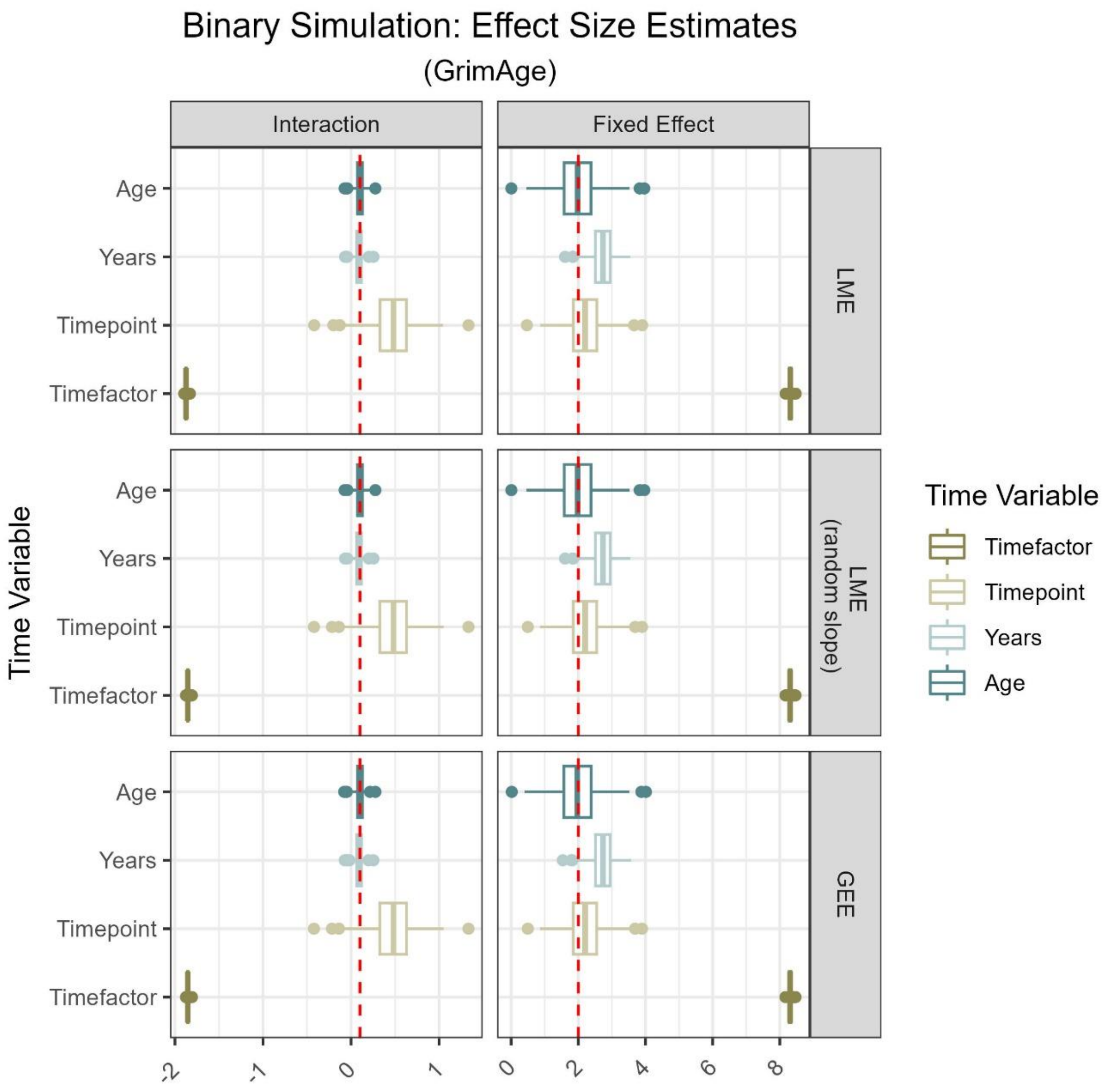

***Figure 19**: Binary exposure simulation results (n = 1,000) based on longitudinal ARIES data (three measurements, age 7, 9, and 15-17)* [1]. *Rows show boxplots of effect size estimates derived from different models and time variables included in those models respectively. The two columns divide into interaction effect (left column) and fixed effect (right column). Simulated interaction effect of 0.1, and simulated fixed effect of 2, are marked in red. Models are Linear Mixed Effect Models (LME) and Generalized Estimating Equations (GEE). Time variables are chronological age (Age), years between measures (Years), number of measure (Timepoint, i.e., 1, 2, 3), factorized measure (Timefactor, i.e., F07, F09, F15). All models contained GrimAge* [3] *derived EA as outcome.*

2. Three Measure-based Simulation

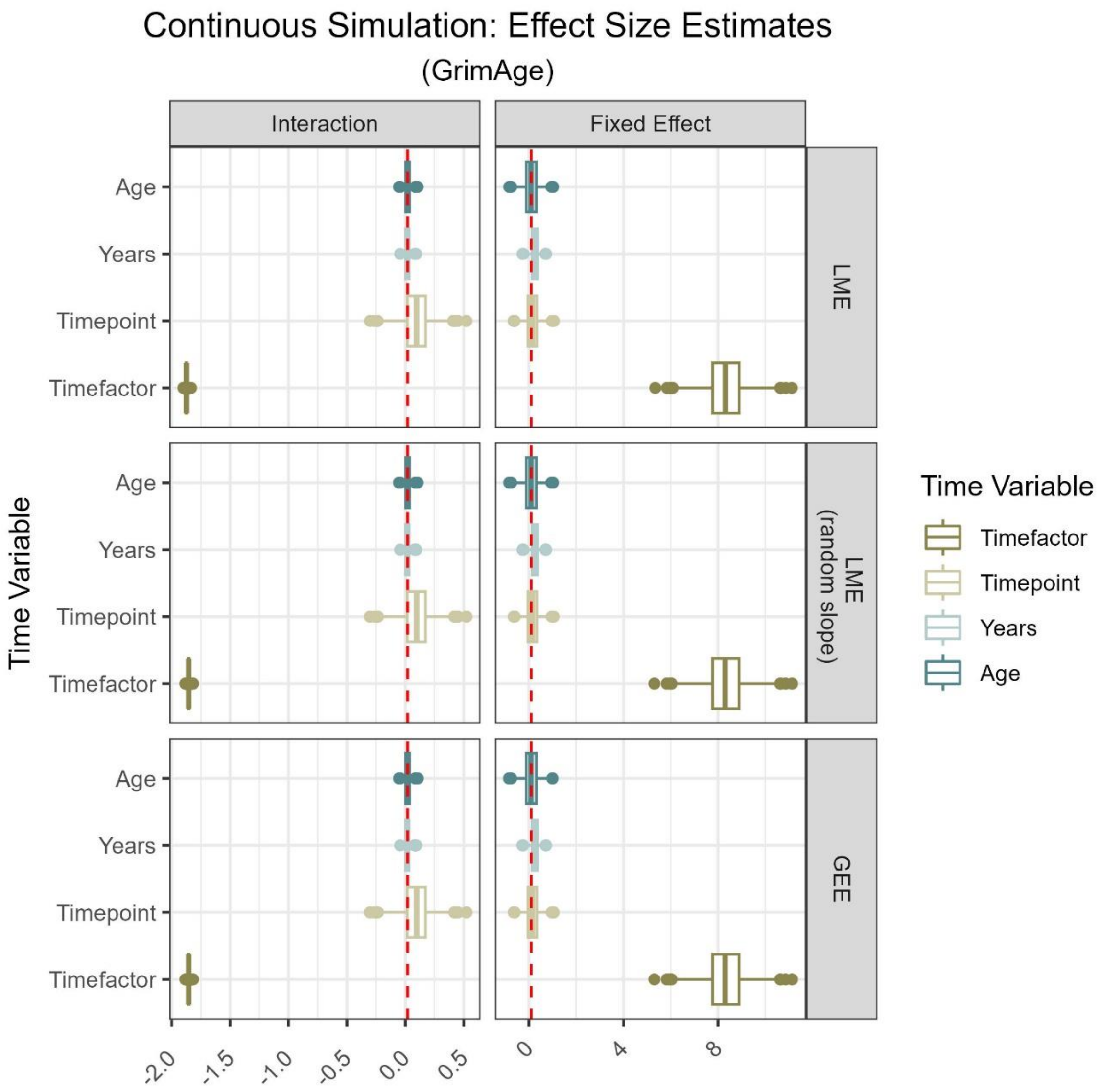

***Figure 20**: Continuous exposure simulation results (n = 1,000) based on longitudinal ARIES data (three measurements, age 7, 9, and 15-17)* [1]. *Rows show boxplots of effect size estimates derived from different models and time variables included in those models respectively. The two columns divide into interaction effect (left column) and fixed effect (right column). Simulated interaction effect of 0.02, and simulated fixed effect of 0.1, are marked in red. Models are Linear Mixed Effect Models (LME) and Generalized Estimating Equations (GEE). Time variables are chronological age (Age), years between measures (Years), number of measure (Timepoint, i.e., 1, 2, 3), factorized measure (Timefactor, i.e., F07, F09, F15). All models contained GrimAge* [3] *derived EA as outcome.*

2. Three Measure-based Simulation

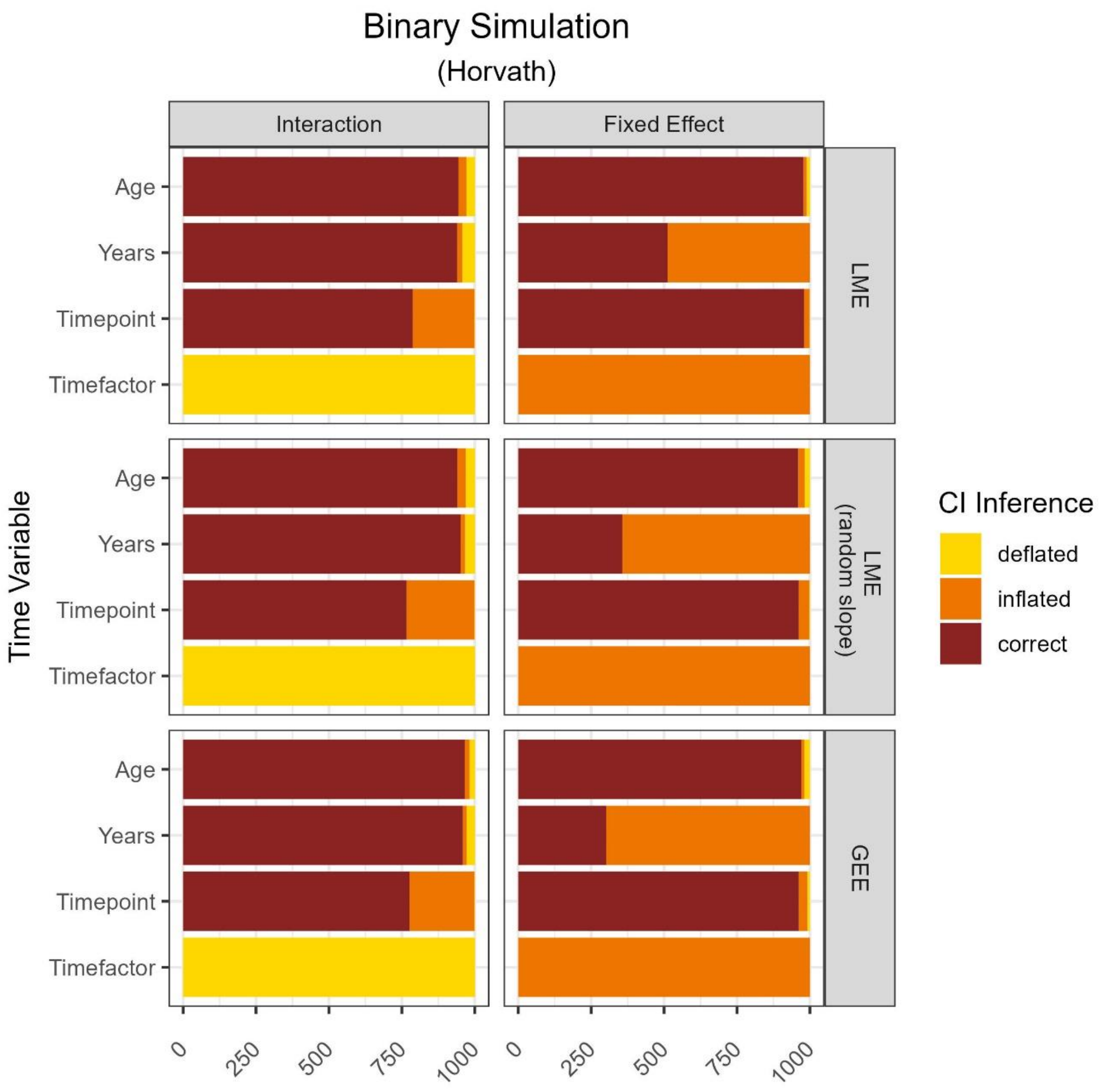

***Figure 21**: Evaluation of confidence intervals (CI) from binary exposure simulation results (n = 1,000) based on longitudinal ARIES data (three measurements, age 7, 9, and 15-17)* [1]*. Rows show the number of simulation results, from different models and time variables, falling in each of the three categories: inflated (CI fully above simulated effect size), deflated (CI fully below simulated effect size), and correct (CI contains simulated effect size). The two columns divide into interaction effect (left column) and fixed effect (right column). Models are Linear Mixed Effect Models (LME) and Generalized Estimating Equations (GEE). Time variables are chronological age (Age), years between measures (Years), number of measure (Timepoint, i.e., 1, 2, 3), factorized measure (Timefactor, i.e., F07, F09, F15). All models contained Horvath clock* [2] *derived EA as outcome.*

2. Three Measure-based Simulation

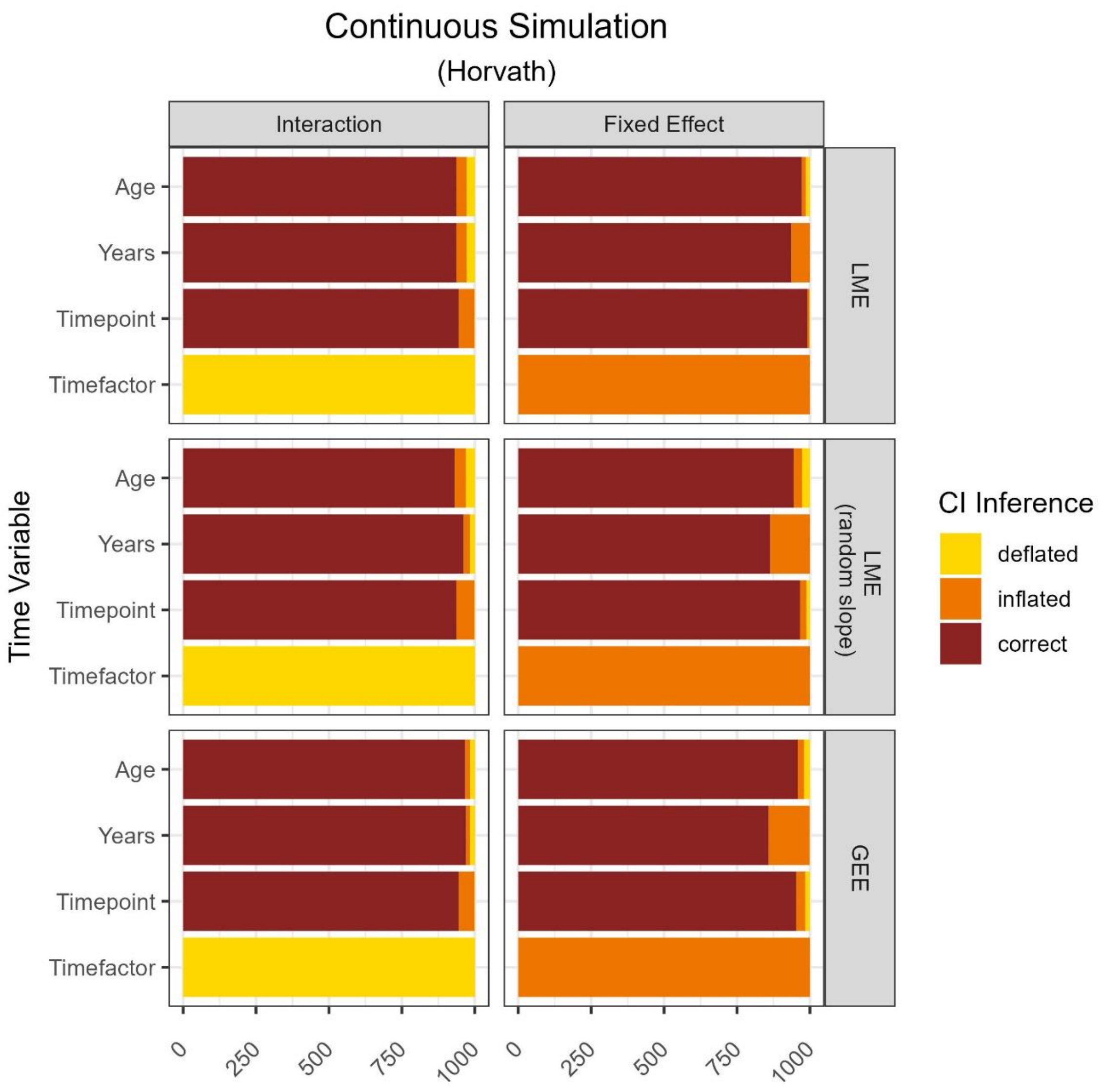

***Figure 22**: Evaluation of confidence intervals (CI) from continuous exposure simulation results (n = 1,000) based on longitudinal ARIES data (three measurements, age 7, 9, and 15-17)* [1]*. Rows show the number of simulation results, from different models and time variables, falling in each of the three categories: inflated (CI fully above simulated effect size), deflated (CI fully below simulated effect size), and correct (CI contains simulated effect size). The two columns divide into interaction effect (left column) and fixed effect (right column). Models are Linear Mixed Effect Models (LME) and Generalized Estimating Equations (GEE). Time variables are chronological age (Age), years between measures (Years), number of measure (Timepoint, i.e., 1, 2, 3), factorized measure (Timefactor, i.e., F07, F09, F15). All models contained Horvath clock* [2] *derived EA as outcome.*

2. Three Measure-based Simulation

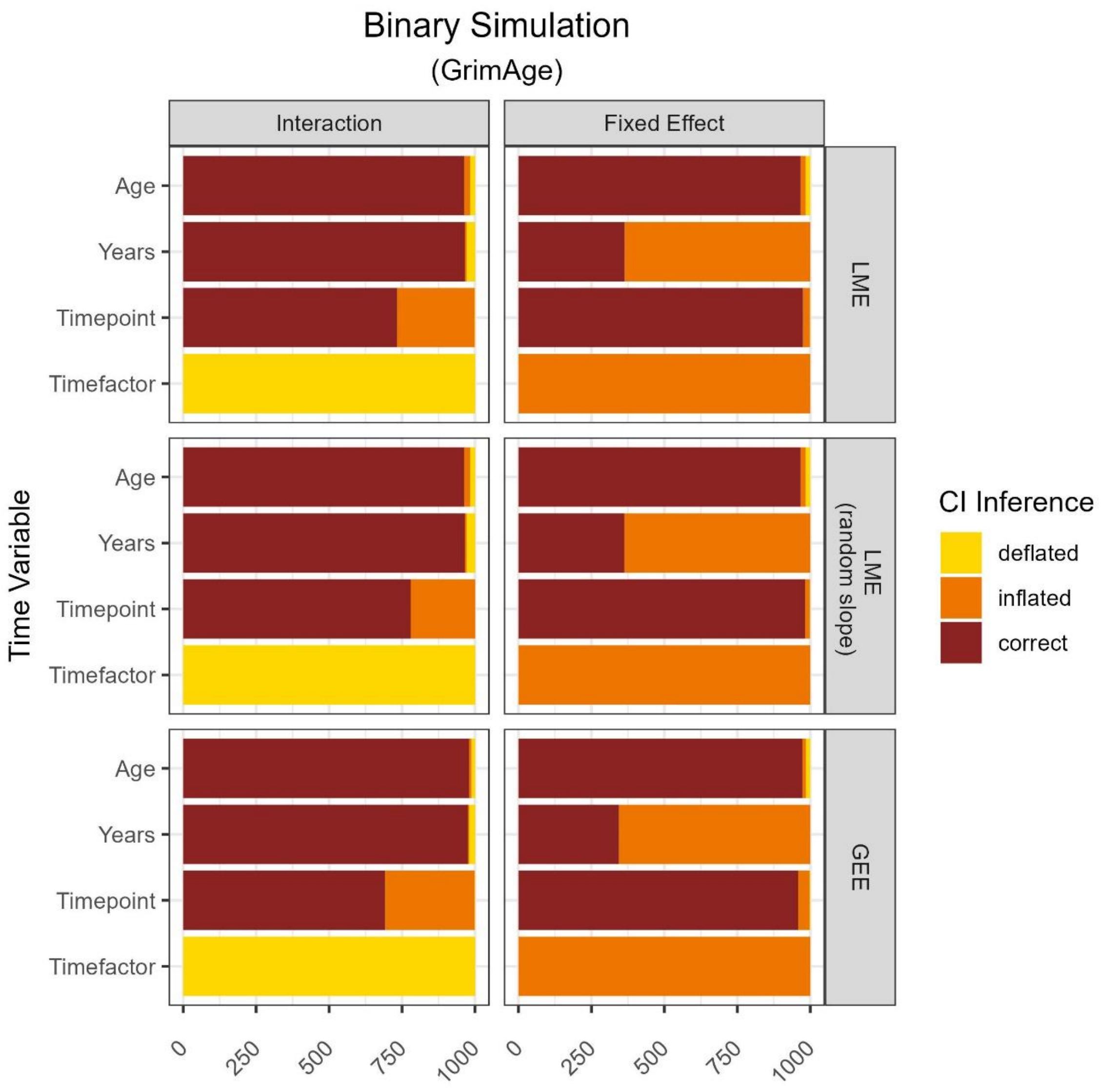

***Figure 23**: Evaluation of confidence intervals (CI) from binary exposure simulation results (n = 1,000) based on longitudinal ARIES data (three measurements, age 7, 9, and 15-17)* [1]*. Rows show the number of simulation results, from different models and time variables, falling in each of the three categories: inflated (CI fully above simulated effect size), deflated (CI fully below simulated effect size), and correct (CI contains simulated effect size). The two columns divide into interaction effect (left column) and fixed effect (right column). Models are Linear Mixed Effect Models (LME) and Generalized Estimating Equations (GEE). Time variables are chronological age (Age), years between measures (Years), number of measure (Timepoint, i.e., 1, 2, 3), factorized measure (Timefactor, i.e., F07, F09, F15). All models contained GrimAge* [3] *derived EA as outcome.*

2. Three Measure-based Simulation

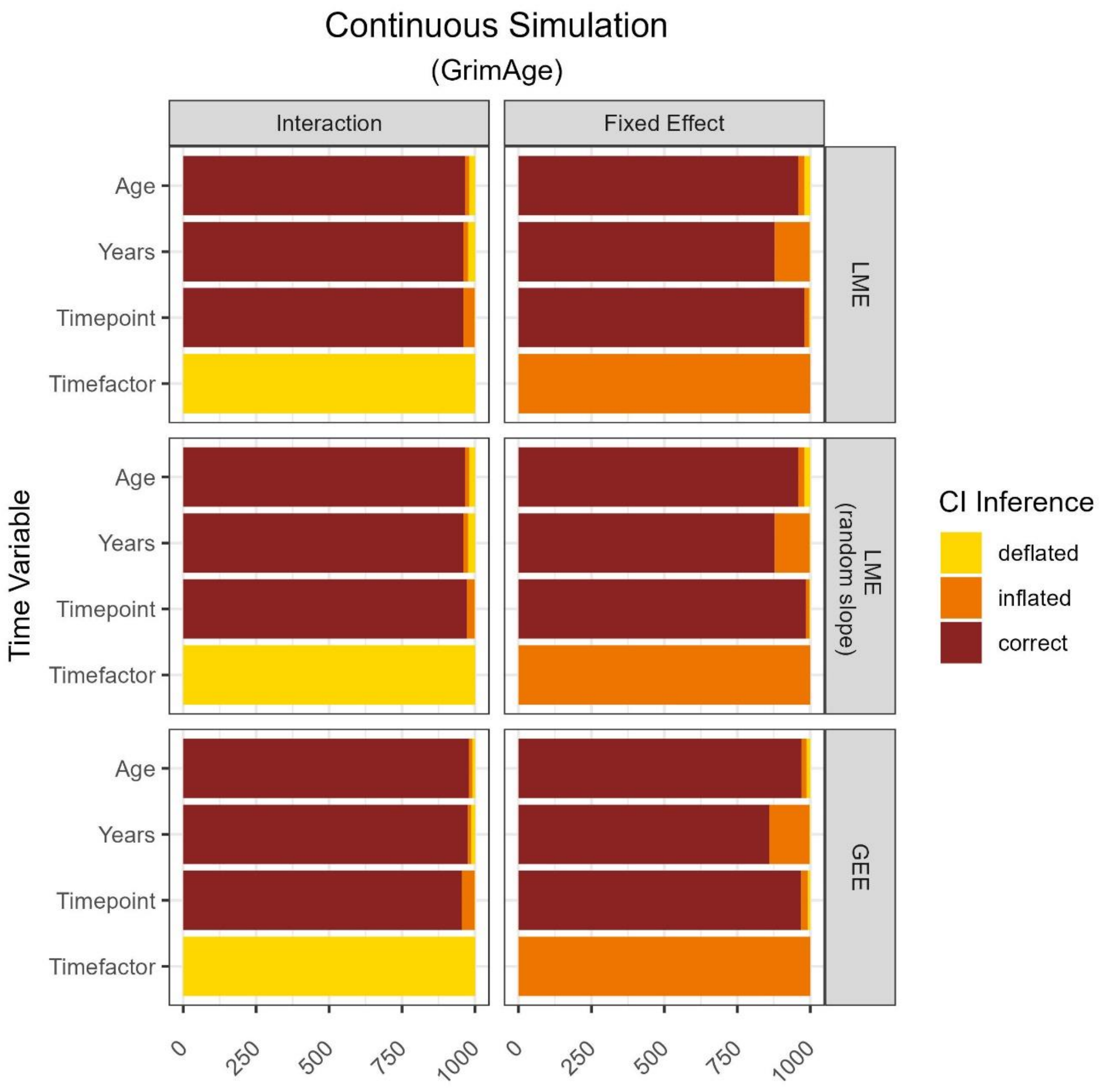

***Figure 24**: Evaluation of confidence intervals (CI) from continuous exposure simulation results (n = 1,000) based on longitudinal ARIES data (three measurements, age 7, 9, and 15-17)* [1]*. Rows show the number of simulation results, from different models and time variables, falling in each of the three categories: inflated (CI fully above simulated effect size), deflated (CI fully below simulated effect size), and correct (CI contains simulated effect size). The two columns divide into interaction effect (left column) and fixed effect (right column). Models are Linear Mixed Effect Models (LME) and Generalized Estimating Equations (GEE). Time variables are chronological age (Age), years between measures (Years), number of measure (Timepoint, i.e., 1, 2, 3), factorized measure (Timefactor, i.e., F07, F09, F15). All models contained GrimAge* [3] *derived EA as outcome.*

3. Real-world Example

***Table 7**: Fixed effect estimates and confidence intervals (CI) from biological sex on longitudinal EA in ARIES cohort data (two measurements, age 7 and 15-17)* [1]. *Methods used to model the effect of both exposures differed in (i) the type of model, (ii) the outcome and (iii) time variables included in the model. Rows show results from each model and time variable. Columns divide results in outcome variables included in the model. Models are Linear Mixed Effect Models (LME), Generalized Estimating Equations (GEE), and regression on difference between two epigenetic age (EA) measures (Δ aging). All models were corrected for cell type proportion, models using birthweight as exposure additionally account for sex. Time variables are chronological age (Age), years between measures (Years), number of measure (Timepoint, i.e., 1, 2, 3), factorized measure (Timefactor, i.e., F07, F09, F15). Outcome variables are EAA (i.e., residual from regressing EA on age), or EA itself, derived from either the Horvath clock* [2], *GrimAge* [3], *or their principal component versions* [4]. *Significant results, with p-values below a threshold of 0.05, were marked with an asterisk.*

		**Fixed Effect Estimates**			
		Exposure: Biological Sex			
**Model**	**Time Variable**	**Outcome**			
		Horvath (EA)	GrimAge (EA)	PC Horvath (EA)	PC GrimAge (EA)
LME	Age	-0.11 (-0.86,0.64)	2.83 (2.39,3.28) *	0.07 (-0.42,0.57)	2.15 (1.86,2.44) *
	Years	0.34 (-0.09,0.77)	2.2 (1.93,2.47) *	0.1 (-0.23,0.44)	1.64 (1.44,1.84) *
	Timepoint	-0.39 (-1.28,0.5)	2.9 (2.35,3.45) *	-0.04 (-0.64,0.56)	2.21 (1.81,2.61) *
	Timefactor	0.71 (0.37,1.05) *	1.84 (1.62,2.07) *	0.18 (-0.12,0.48)	1.35 (1.16,1.55) *
LME (random slope)	Age	-0.12 (-0.72,0.47)	2.83 (2.41,3.26) *	0.1 (-0.27,0.46)	2.15 (1.88,2.42) *
	Years	0.36 (0.05,0.66) *	2.2 (1.95,2.45) *	0.11 (-0.14,0.36)	1.64 (1.45,1.83) *
	Timepoint	-0.36 (-1.07,0.36)	2.92 (2.41,3.43) *	-0.02 (-0.46,0.42)	2.21 (1.88,2.55) *
	Timefactor	0.71 (0.37,1.05) *	1.84 (1.62,2.07) *	0.18 (-0.12,0.48)	1.35 (1.16,1.55) *
GEE	Age	-0.09 (-0.9,0.72)	2.82 (2.32,3.33) *	0.13 (-0.5,0.77)	2.09 (1.71,2.47) *
	Years	0.33 (-0.09,0.76) *	2.2 (1.93,2.46) *	0.13 (-0.2,0.47)	1.62 (1.42,1.83) *
	Timepoint	-0.39 (-1.36,0.57)	2.89 (2.27,3.51) *	0.04 (-0.73,0.8)	2.18 (1.68,2.68) *
	Timefactor	0.69 (0.38,1.01) *	1.84 (1.64,2.04) *	0.19 (-0.06,0.44)	1.35 (1.19,1.51) *

3. Real-world Example

***Table 8**: Interaction effect estimates and confidence intervals (CI) from biological sex on longitudinal EA in ARIES cohort data (two measurements, age 7 and 15-17)* [1]. *Methods used to model the effect of both exposures differed in (i) the type of model, (ii) the outcome and (iii) time variables included in the model. Rows show results from each model and time variable. Columns divide results in outcome variables included in the model. Models are Linear Mixed Effect Models (LME), Generalized Estimating Equations (GEE), and regression on difference between two epigenetic age (EA) measures (Δ aging). All models were corrected for cell type proportion, models using birthweight as exposure additionally account for sex. Time variables are chronological age (Age), years between measures (Years), number of measure (Timepoint, i.e., 1, 2, 3), factorized measure (Timefactor, i.e., F07, F09, F15). Outcome variables are EAA (i.e., residual from regressing EA on age), or EA itself, derived from either the Horvath clock* [2], *GrimAge* [3], *or their principal component versions* [4]. *Significant results, with p-values below a threshold of 0.05, were marked with an asterisk.*

		**Interaction Effect Estimates**			
		Exposure: Biological Sex			
**Model**	**Time Variable**	**Outcome**			
		Horvath (EA)	GrimAge (EA)	PC Horvath (EA)	PC GrimAge (EA)
LME	Age	0.07 (0.01,0.12) *	-0.08 (-0.11,-0.05) *	0.01 (-0.02,0.04)	-0.07 (-0.08,-0.05) *
	Years	0.07 (0.01,0.12) *	-0.07 (-0.1,-0.04) *	0.01 (-0.02,0.04)	-0.06 (-0.08,-0.04) *
	Timepoint	0.73 (0.18,1.28) *	-0.71 (-1.04,-0.37) *	0.15 (-0.2,0.5)	-0.57 (-0.81,-0.34) *
	Timefactor	0.52 (0.13,0.91) *	-0.5 (-0.74,-0.26) *	0.1 (-0.14,0.35)	-0.4 (-0.57,-0.24) *
LME (random slope)	Age	0.07 (0.02,0.12) *	-0.08 (-0.11,-0.05) *	0 (-0.03,0.04)	-0.07 (-0.08,-0.05) *
	Years	0.06 (0.01,0.12) *	-0.07 (-0.1,-0.04) *	0.01 (-0.03,0.04)	-0.06 (-0.08,-0.04) *
	Timepoint	0.72 (0.23,1.21) *	-0.72 (-1.06,-0.39) *	0.12 (-0.22,0.45)	-0.57 (-0.81,-0.34) *
	Timefactor	0.52 (0.13,0.91) *	-0.5 (-0.74,-0.26) *	0.1 (-0.14,0.35)	-0.4 (-0.57,-0.24) *
GEE	Age	0.06 (0,0.12) *	-0.08 (-0.12,-0.04) *	0 (-0.04,0.05)	-0.06 (-0.09,-0.03) *
	Years	0.06 (0.01,0.12) *	-0.07 (-0.11,-0.04) *	0.01 (-0.04,0.05)	-0.06 (-0.08,-0.03) *
	Timepoint	0.72 (0.11,1.34) *	-0.7 (-1.09,-0.31) *	0.1 (-0.38,0.59)	-0.55 (-0.87,-0.23) *
	Timefactor	0.51 (0.08,0.95) *	-0.5 (-0.77,-0.22) *	0.07 (-0.27,0.42)	-0.39 (-0.62,-0.17) *
Δ aging	Age	0.07 (0.01,0.13) *	-0.1 (-0.14,-0.06) *	0.02 (-0.03,0.06)	-0.09 (-0.12,-0.06) *
	None	0.74 (0.16,1.31) *	-0.97 (-1.37,-0.58) *	0.27 (-0.21,0.74)	-0.86 (-1.21,-0.52) *

3. Real-world Example

Table 9*: Fixed effect estimates and confidence intervals (CI) from birthweight on longitudinal EA in ARIES cohort data (two measurements, age 7 and 15-17)* [1]. *Methods used to model the effect of both exposures differed in (i) the type of model, (ii) the outcome and (iii) time variables included in the model. Rows show results from each model and time variable. Columns divide results in outcome variables included in the model. Models are Linear Mixed Effect Models (LME), Generalized Estimating Equations (GEE), and regression on difference between two epigenetic age (EA) measures (Δ aging). All models were corrected for cell type proportion, models using birthweight as exposure additionally account for sex. Time variables are chronological age (Age), years between measures (Years), number of measure (Timepoint, i.e., 1, 2, 3), factorized measure (Timefactor, i.e., F07, F09, F15). Outcome variables are EAA (i.e., residual from regressing EA on age), or EA itself, derived from either the Horvath clock* [2], *GrimAge* [3], *or their principal component versions* [4]. *Significant results, with p-values below a threshold of 0.05, were marked with an asterisk.*

		**Fixed Effect Estimates**			
		Exposure: Birthweight			
**Model**	**Time Variable**	**Outcome**			
		Horvath (EA)	GrimAge (EA)	PC Horvath (EA)	PC GrimAge (EA)
LME	Age	1.09 (0.33,1.86) *	-0.2 (-0.66,0.26)	0.1 (-0.41,0.61)	-0.41 (-0.71,-0.1) *
	Years	0.41 (-0.03,0.84)	-0.24 (-0.52,0.03)	-0.09 (-0.43,0.25)	-0.34 (-0.55,-0.13) *
	Timepoint	1.29 (0.38,2.2) *	-0.22 (-0.79,0.35)	0.14 (-0.47,0.75)	-0.47 (-0.88,-0.05) *
	Timefactor	-0.03 (-0.37,0.32)	-0.27 (-0.5,-0.04) *	-0.22 (-0.52,0.09)	-0.3 (-0.49,-0.1) *
LME (random slope)	Age	1.08 (0.48,1.69) *	-0.21 (-0.65,0.22)	0.1 (-0.28,0.47)	-0.43 (-0.71,-0.15) *
	Years	0.43 (0.11,0.74) *	-0.25 (-0.51,0)	-0.09 (-0.34,0.17)	-0.36 (-0.55,-0.17) *
	Timepoint	1.3 (0.56,2.03) *	-0.23 (-0.75,0.29)	0.15 (-0.3,0.6)	-0.49 (-0.83,-0.14) *
	Timefactor	-0.03 (-0.37,0.32)	-0.27 (-0.5,-0.04) *	-0.22 (-0.52,0.09)	-0.3 (-0.49,-0.1) *
GEE	Age	1.12 (0.29,1.95) *	-0.19 (-0.71,0.32)	0.2 (-0.45,0.84)	-0.4 (-0.79,-0.01) *
	Years	0.42 (-0.02,0.85) *	-0.25 (-0.52,0.02)	-0.06 (-0.4,0.28)	-0.34 (-0.55,-0.13) *
	Timepoint	1.3 (0.31,2.3) *	-0.22 (-0.86,0.41)	0.22 (-0.56,1.01)	-0.48 (-0.99,0.03) *
	Timefactor	-0.03 (-0.34,0.29)	-0.28 (-0.49,-0.08) *	-0.23 (-0.48,0.02)	-0.31 (-0.47,-0.14) *

3. Real-world Example

Table 10*: Interaction effect estimates and confidence intervals (CI) from birthweight on longitudinal EA in ARIES cohort data (two measurements, age 7 and 15-17)* [1]. *Methods used to model the effect of both exposures differed in (i) the type of model, (ii) the outcome and (iii) time variables included in the model. Rows show results from each model and time variable. Columns divide results in outcome variables included in the model. Models are Linear Mixed Effect Models (LME), Generalized Estimating Equations (GEE), and regression on difference between two epigenetic age (EA) measures (Δ aging). All models were corrected for cell type proportion, models using birthweight as exposure additionally account for sex. Time variables are chronological age (Age), years between measures (Years), number of measure (Timepoint, i.e., 1, 2, 3), factorized measure (Timefactor, i.e., F07, F09, F15). Outcome variables are EAA (i.e., residual from regressing EA on age), or EA itself, derived from either the Horvath clock* [2], *GrimAge* [3], *or their principal component versions* [4]. *Significant results, with p-values below a threshold of 0.05, were marked with an asterisk.*

		**Interaction Effect Estimates**			
		Exposure: Birthweight			
**Model**	**Time Variable**	**Outcome**			
		Horvath (EA)	GrimAge (EA)	PC Horvath (EA)	PC GrimAge (EA)
LME	Age	-0.09 (-0.15,-0.03) *	-0.01 (-0.04,0.03)	-0.02 (-0.06,0.01)	0.01 (-0.01,0.03)
	Years	-0.08 (-0.14,-0.03) *	-0.01 (-0.04,0.02)	-0.03 (-0.06,0.01)	0.01 (-0.01,0.03)
	Timepoint	-0.88 (-1.44,-0.31) *	-0.03 (-0.38,0.32)	-0.24 (-0.59,0.12)	0.11 (-0.13,0.35)
	Timefactor	-0.62 (-1.02,-0.22) *	-0.02 (-0.27,0.22)	-0.17 (-0.42,0.08)	0.08 (-0.09,0.25)
LME (random slope)	Age	-0.09 (-0.14,-0.04) *	0 (-0.04,0.03)	-0.02 (-0.06,0.01)	0.01 (-0.01,0.03)
	Years	-0.09 (-0.14,-0.03) *	-0.01 (-0.04,0.03)	-0.03 (-0.06,0.01)	0.01 (-0.01,0.03)
	Timepoint	-0.87 (-1.37,-0.37) *	-0.03 (-0.38,0.31)	-0.24 (-0.58,0.09)	0.11 (-0.13,0.35)
	Timefactor	-0.62 (-1.02,-0.22) *	-0.02 (-0.27,0.22)	-0.17 (-0.42,0.08)	0.08 (-0.09,0.25)
GEE	Age	-0.09 (-0.16,-0.03) *	-0.01 (-0.05,0.03)	-0.03 (-0.08,0.02)	0.01 (-0.02,0.04)
	Years	-0.08 (-0.14,-0.03) *	-0.01 (-0.04,0.03)	-0.03 (-0.08,0.01)	0.01 (-0.02,0.03)
	Timepoint	-0.89 (-1.51,-0.26) *	-0.04 (-0.44,0.36)	-0.3 (-0.8,0.19)	0.11 (-0.21,0.44)
	Timefactor	-0.63 (-1.07,-0.18) *	-0.03 (-0.31,0.26)	-0.21 (-0.57,0.14)	0.08 (-0.15,0.31)
Δ aging	Age	-0.1 (-0.16,-0.04) *	0 (-0.04,0.04)	-0.03 (-0.08,0.02)	0 (-0.03,0.04)
	None	-0.98 (-1.58,-0.39) *	-0.06 (-0.47,0.35)	-0.29 (-0.79,0.21)	0.02 (-0.35,0.38)

3. Real-world Example

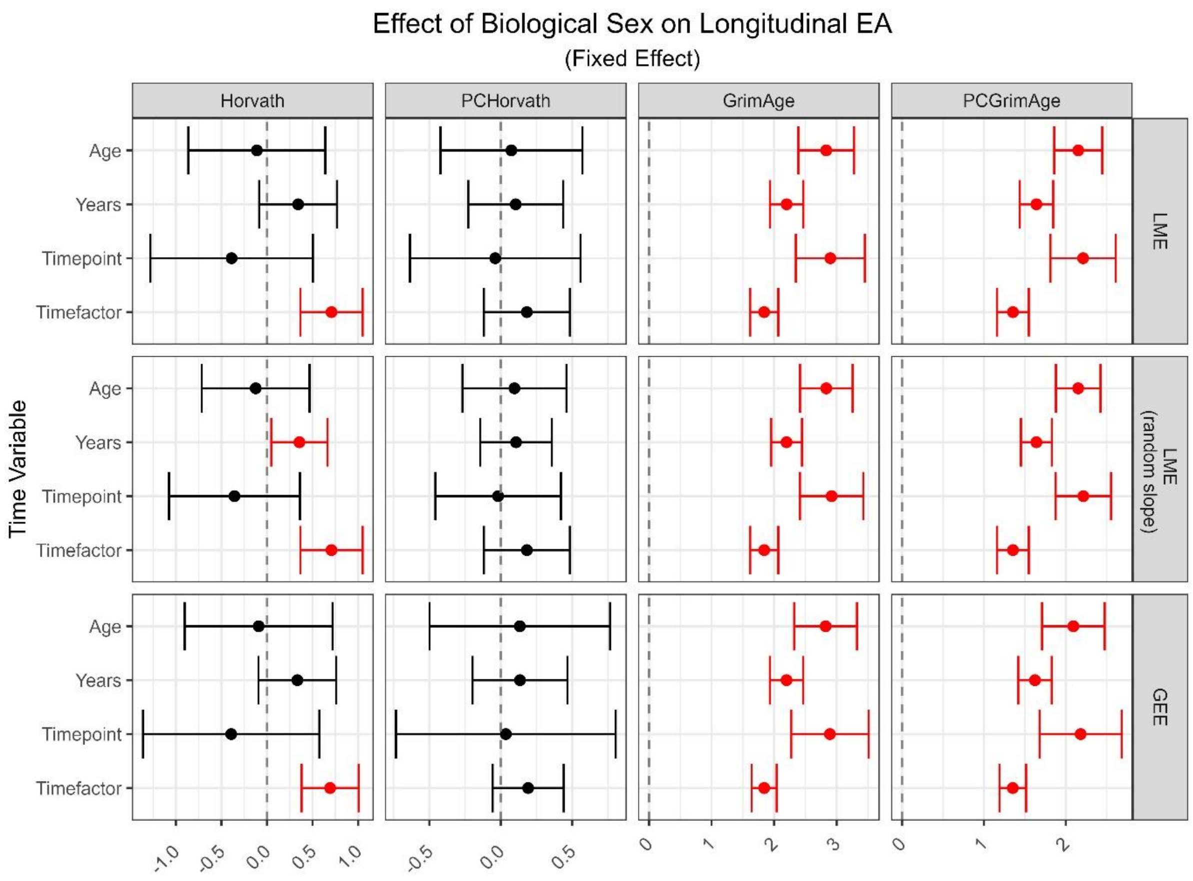

***Figure 25**: Fixed effect estimates of biological sex on offspring EA over time. Included were two within-person measures (age 7 and 15-17) from ARIES* [1]. *Rows contain effect size estimates as well as confidence intervals derived from different models and time variables included in those models respectively. Effect estimates are based on females as reference. Significant estimates (significance level 0.05) are marked in red. Models are Linear Mixed Effect Models (LME), Generalized Estimating Equations (GEE), and regression on difference between two epigenetic age (EA) measures (Δ aging). Time variables are chronological age (Age), years between measures (Years), number of measure (Timepoint, i.e., 1, 2, 3), factorized measure (Timefactor, i.e., F07, F09, F15). All models contained EA as outcome, derived from either the Horvath clock* [2], *GrimAge* [3], *or their principal component versions* [4], *and were adjusted for cell type proportions.*

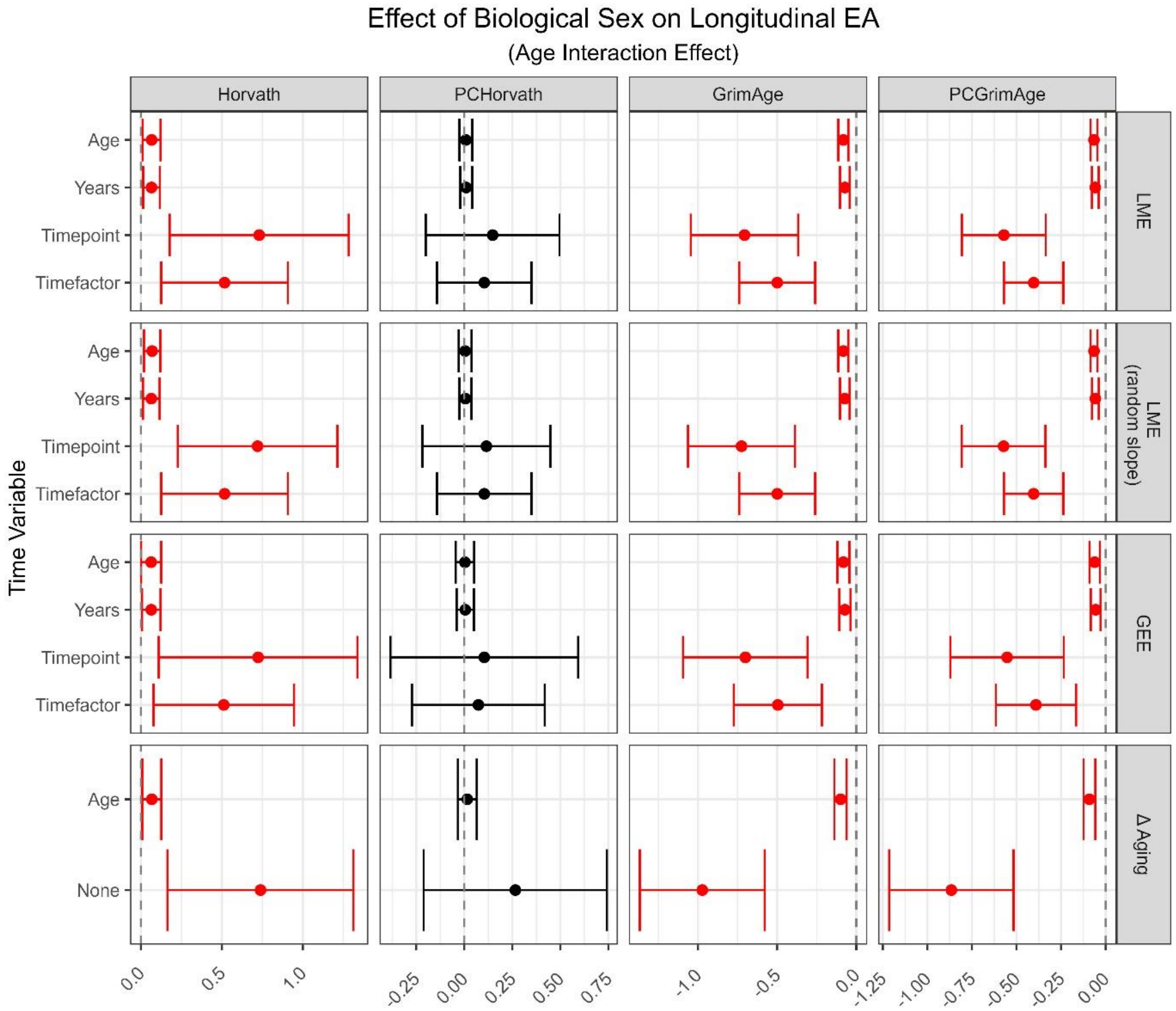

***Figure 26**: Age interaction estimates of biological sex on offspring EA over time. Included were two within-person measures (age 7 and 15-17) from ARIES* [1]. *Rows contain effect size estimates as well as confidence intervals derived from different models and time variables included in those models respectively. Significant estimates (significance level 0.05) are marked in red. Models are Linear Mixed Effect Models (LME), Generalized Estimating Equations (GEE), and regression on difference between two epigenetic age (EA) measures (Δ aging). Time variables are chronological age (Age), years between measures (Years), number of measure (Timepoint, i.e., 1, 2, 3), factorized measure (Timefactor, i.e., F07, F09, F15). All models contained EA as outcome, derived from either the Horvath clock* [2], *GrimAge* [3], *or their principal component versions* [4], *and were adjusted for cell type proportions.*

3. Real-world Example

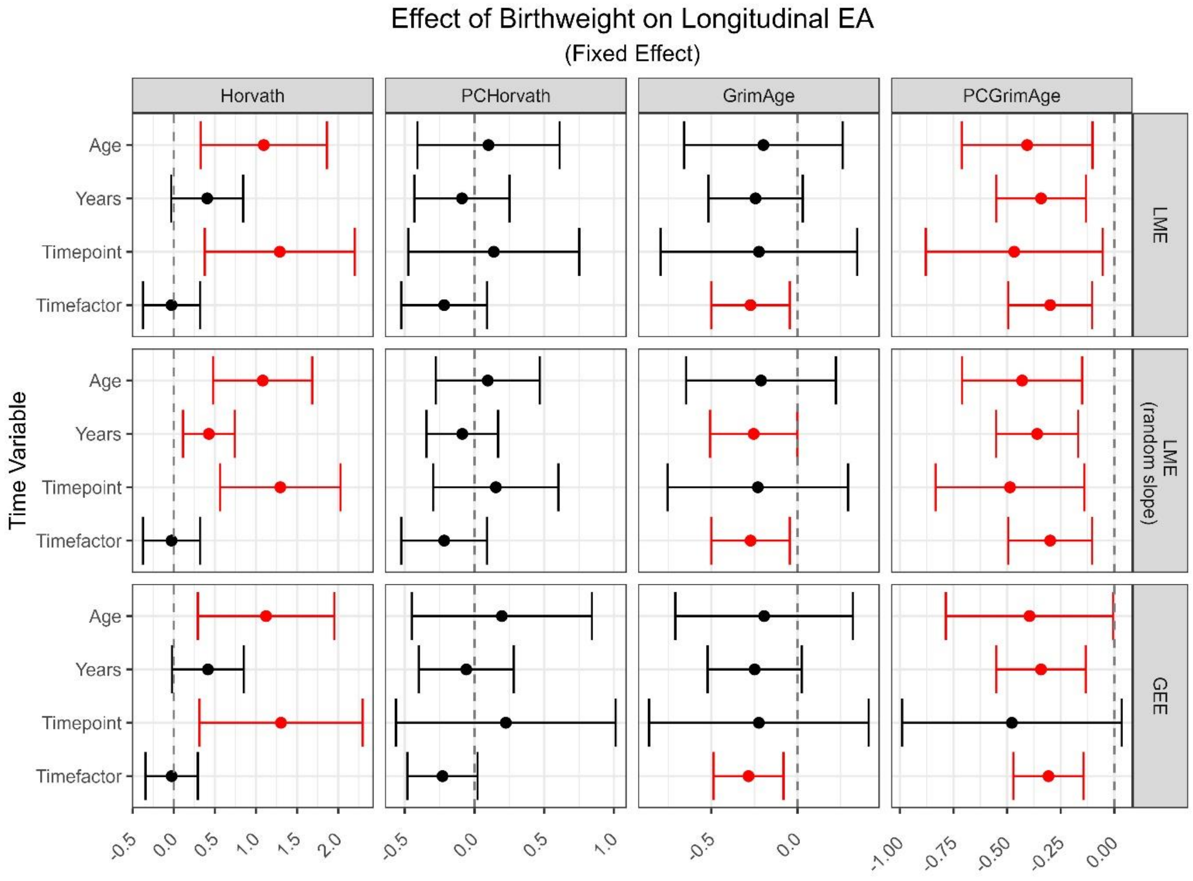

***Figure 27**: Fixed effect estimates of birthweight on offspring EA over time. Included were two within-person measures (age 7 and 15-17) from ARIES* [1]*. Rows contain effect size estimates as well as confidence intervals derived from different models and time variables included in those models respectively. Effect estimates are based on females as reference. Significant estimates (significance level 0.05) are marked in red. Models are Linear Mixed Effect Models (LME), Generalized Estimating Equations (GEE), and regression on difference between two epigenetic age (EA) measures (Δ aging). Time variables are chronological age (Age), years between measures (Years), number of measure (Timepoint, i.e., 1, 2, 3), factorized measure (Timefactor, i.e., F07, F09, F15). All models contained EA as outcome, derived from either the Horvath clock* [2]*, GrimAge* [3]*, or their principal component versions* [4]*, and were adjusted for biological sex and cell type proportions.*

3. Real-world Example

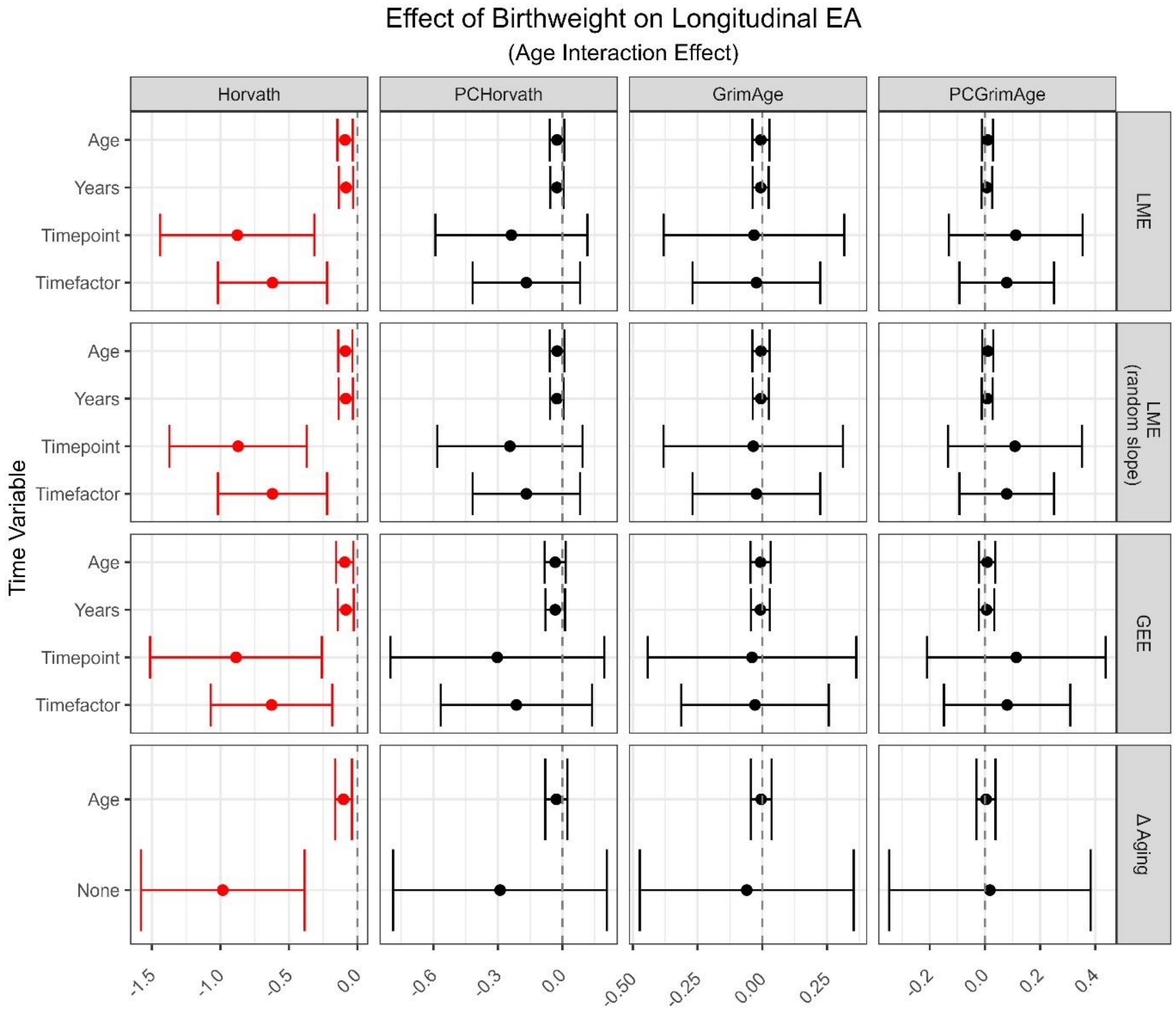

***Figure 28**: Age interaction estimates of birthweight on offspring EA over time. Included were two within-person measures (age 7 and 15-17) from ARIES* [1]. *Rows contain effect size estimates as well as confidence intervals derived from different models and time variables included in those models respectively. Significant estimates (significance level 0.05) are marked in red. Models are Linear Mixed Effect Models (LME), Generalized Estimating Equations (GEE), and regression on difference between two epigenetic age (EA) measures (Δ aging). Time variables are chronological age (Age), years between measures (Years), number of measure (Timepoint, i.e., 1, 2, 3), factorized measure (Timefactor, i.e., F07, F09, F15). All models contained EA as outcome, derived from either the Horvath clock* [2], *GrimAge* [3], *or their principal component versions* [4], *and were adjusted for biological sex and cell type proportions.*

References

1. Relton CL, Gaunt T, McArdle W, Ho K, Duggirala A, Shihab H, et al. Data resource profile: Accessible resource for integrated epigenomic studies (ARIES). Int J Epidemiol. 2015;44:1181–90.

2. Horvath S. DNA methylation age of human tissues and cell types. Genome Biol. 2013;14.

3. Lu AT, Quach A, Wilson JG, Reiner AP, Aviv A, Raj K, et al. DNA methylation GrimAge strongly predicts lifespan and healthspan. Aging (Albany NY). 2019;11:303.

4. Higgins-Chen AT, Thrush KL, Wang Y, Minteer CJ, Kuo PL, Wang M, et al. A computational solution for bolstering reliability of epigenetic clocks: Implications for clinical trials and longitudinal tracking. Nat aging. 2022;2:644.
